# A bird-like skull in a Triassic diapsid reptile increases heterogeneity of the morphological and phylogenetic radiation of Diapsida

**DOI:** 10.1098/rsos.170499

**Published:** 2017-10-11

**Authors:** Adam C. Pritchard, Sterling J. Nesbitt

**Affiliations:** 1 Department of Anatomical Sciences, Stony Brook University, Stony Brook, NY 11794, USA; 2 Department of Geology and Geophysics, Yale University, 210 Whitney Avenue, New Haven, CT 06520-8109, USA; 3 Department of Geosciences, Virginia Tech, Blacksburg, VA 24061, USA

**Keywords:** Reptilia, phylogeny, convergence, Permo-Triassic extinction, evolutionary radiation, Triassic

## Abstract

The Triassic Period saw the first appearance of numerous amniote lineages (e.g. Lepidosauria, Archosauria, Mammalia) that defined Mesozoic ecosystems following the end Permian Mass Extinction, as well as the first major morphological diversification of crown-group reptiles. Unfortunately, much of our understanding of this event comes from the record of large-bodied reptiles (total body length > 1 m). Here we present a new species of drepanosaurid (small-bodied, chameleon-like diapsids) from the Upper Triassic Chinle Formation of New Mexico. Using reconstructions of micro-computed tomography data, we reveal the three-dimensional skull osteology of this clade for the first time. The skull presents many archaic anatomical traits unknown in Triassic crown-group reptiles (e.g. absence of bony support for the external ear), whereas other traits (e.g. toothless rostrum, anteriorly directed orbits, inflated endocranium) resemble derived avian theropods. A phylogenetic analysis of Permo-Triassic diapsids supports the hypothesis that drepanosaurs are an archaic lineage that originated in the Permian, far removed from crown-group Reptilia. The phylogenetic position of drepanosaurids indicates the presence of archaic Permian clades among Triassic small reptile assemblages and that morphological convergence produced a remarkably bird-like skull nearly 100 Myr before one is known to have emerged in Theropoda.

## 1. Background

The Triassic has long been recognized as a critical interval in the history of vertebrate life, especially in terms of the diversification of important Mesozoic taxa. It saw the global recovery from the biodiversity crash of the Permo-Triassic Extinction (PTE), and the first appearances of major diapsid reptile clades that would typify Mesozoic ecosystems (e.g. Dinosauromorpha, Lepidosauria, Pseudosuchia, Pterosauria, Ichthyosauria Sauropterygia) [1–7].

However, it has recently been recognized that the morphological diversification of Diapsida in the Triassic was far broader than previously understood [8–11]. A number of bauplans long considered to be restricted to later Mesozoic diapsids are now known in unrelated Triassic lineages. These include bipedal toothless pseudosuchians closely resembling Cretaceous ornithomimosaurs [12], dome-skulled forms similar to pachycephalosaurs [13], pseudosuchian predators with high and narrow skulls similar to large neotheropods [14,15] and a number of long-snouted semiaquatic lineages similar to later neosuchian crocodylomorphs [16–18]. Thus, not only were major taxonomic categories established during the Triassic Period, but suites of morphological features—suites which would typify many Mesozoic and Cenozoic diapsid reptile clades—emerged in a variety of unrelated Triassic species.

As yet, this pattern of convergent morphologies is well established in archosaurs and their close relatives, a possible consequence of overall larger body size and higher preservational potential [19,20]. However, the Triassic Period fossil record rarely preserves small-bodied taxa and critical details of their anatomy that illuminate both their phylogenetic relationships and functional anatomy are lacking [21–23]. Among the most diverse and speciose of these small-bodied lineages are Drepanosauromorpha, a clade of superficially lizard-like diapsids that have been favourably compared with extant arboreal, swimming and burrowing tetrapods [24–28]. Some recent studies support an arboreal habitus for most drepanosauromorphs, including the eponymous *Drepanosaurus* [11,27,29]. Known drepanosauromorph skulls and skeletons are almost all heavily compressed, completely obscuring the three-dimensional skeletal anatomy. The few three-dimensionally preserved specimens are highly incomplete [30,31]. Hypotheses for the phylogenetic affinities of drepanosauromorphs include placement within Lepidosauromorpha [25,30,32], Archosauromorpha [27,28,33,34] and outside of the crown-group reptile clade [35,36]. These are reviewed in appendix A.

**Figure 1.**
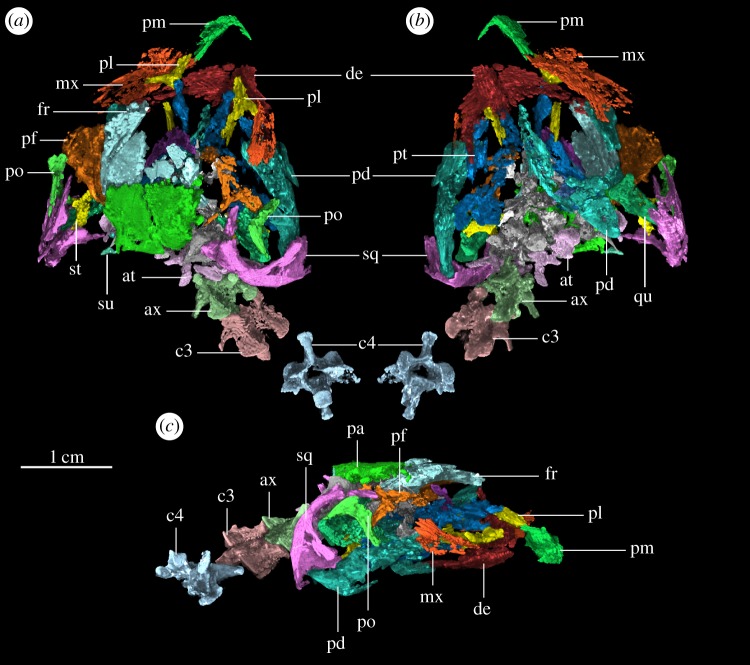
Three-dimensional volume rendering of the *in situ* skull of *Avicranium renestoi* (AMNH FARB 30834) from µCT data in (*a*) dorsal view, (*b*) ventral view and (*c*) left lateral view. Abbreviations: at, atlantal neural arch; ax, axis; c3, cervical vertebra 3; c4, cervical vertebra 4; de, dentary; fr, frontal; mx, maxilla; pd, postdentary elements; pf, postfrontal; pl, palatine; pm, premaxilla; po, postorbital; pt, pterygoid; qu, quadrate; sq, squamosal; su, supratemporal.

**Figure 2.**
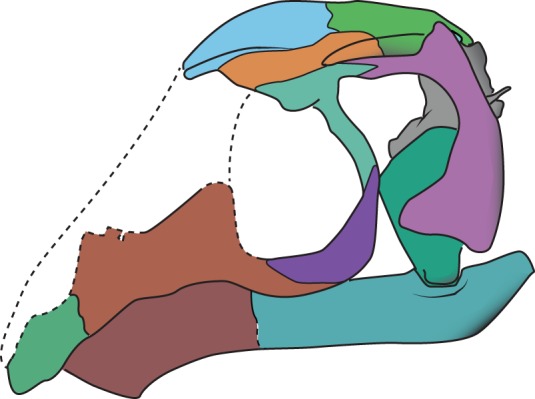
Line drawing of the restored skull of *Avicranium renestoi* based on the three-dimensional surface renderings of skull elements in AMNH FARB 30834.

**Figure 3.**
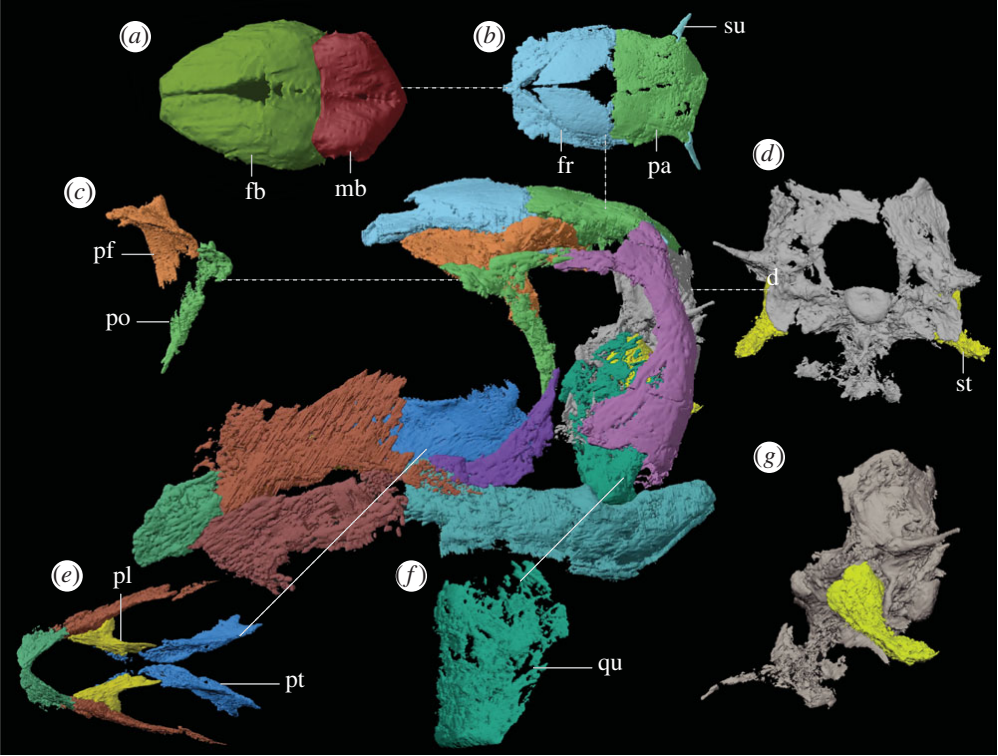
Reconstructed skull of *Avicranium renestoi* based on rearticulated three-dimensional surface rendering of the skull bones of AMNH FARB 30834. Callouts include (*a*) reconstructed endocast in dorsal view, (*b*) skull roof in dorsal view, (*c*) postorbital complex (consisting of postfrontal and postorbital) in anterior view, (*d*) braincase and stapes in posterior view, (*e*) palatal complex in ventral view, (*f*) left quadrate in posterior view and (*g*) braincase and stapes in left lateral view. All bones have been rearticulated based on the facets of the reconstructed elements. Abbreviations: fb, forebrain; mb, midbrain; pa, parietal; pf, postfrontal; pl, palatine; po, postorbital; pt, pterygoid; qu, quadrate; st, stapes; su, supratemporal.

Here, we report on a nearly complete and three-dimensionally preserved skull and partial cervical series of a drepanosauromorph (figure 1) from the Upper Triassic (Late Norian to Rhaetian) Coelophysis Quarry (‘upper sandstone member’, Chinle Formation). Using micro-computed tomography (µCT) scans and three-dimensional modelling, we present a reconstruction of the skull of this new taxon, the first for any drepanosauromorph (figures 1–3; parameters for µCT scanning and reconstruction in appendix B). The quality of preservation allows us to reassess the phylogenetic affinities of Drepanosauromorpha using a data matrix focused on Permo-Triassic diapsids and early Sauria.

## 2. Systematic palaeontology

Diapsida [37]; Drepanosauromorpha [27]; Drepanosauridae [38]; *Avicranium renestoi*, n. gen., n. sp.

### 2.1. Etymology

*Avicranium*, from *aves* (Latin for bird) and *cranium* (Latin for cranium), in reference to the suite of bird-like morphologies present in the holotype skull; *renestoi*, for Silvio Renesto, who described much of the drepanosauromorph fossil record from Triassic Italy.

### 2.2. Holotype

AMNH FARB 30834, partial skull and articulated cervical series. Additional drepanosaurid caudal vertebrae and limb fragments are preserved in the block, but are not clearly associated with the individual to which the skull and cervical vertebrae belong.

### 2.3. Locality

Coelophysis Quarry (‘siltstone member’, Chinle Formation). Recovered during preparation of the holotype block of the shuvosaurid pseudosuchian *Effigia okeeffeae* by S.J.N. [12].

### 2.4. Diagnosis

Specimens for anatomical comparisons are listed in appendix C. A drepanosaurid diapsid differing from *Hypuronector limnaios*, *Megalancosaurus preonensis* and *Vallesaurus cenensis* (the only drepanosauromorphs with skull material) in the complete absence of teeth, a dorsoventrally taller retroarticular process with a triangular shape in lateral view, and cervical neural spines with subequal anteroposterior lengths and transverse widths.

## 3. Comparative anatomy

The identification of this specimen as a drepanosaurid is based on its cervical vertebral anatomy. Drepanosaurids possess heterocoelous cervical vertebral centra with saddle-shaped articular surfaces. The prezygapophyseal facets face anteriorly and extend far anteriorly relative to the anterior margin of the centrum. The neural spines are anteroposteriorly short and strongly inclined anterodorsally. In each of these features, *Av. renestoi* is very similar to drepanosaurids with cervical series, specifically *Drepanosaurus unguicaudatus* [27,31]. The bones of the skull are loosely articulated with one another, much as in the few other known drepanosauromorph skulls. It is similar in size to the known skulls of *Me. preonensis* (approx. 27 mm) and substantially larger than the skull of the holotype of *V. cenensis* (approx. 16 mm).

### 3.1. Bird-like traits

The skull of *Av. renestoi* exhibits a number of striking similarities to avian theropods (figures 2 and 3). The rostrum is slender and acuminate, as has been noted in the Italian drepanosaurid *Me. preonensis* [39,40]. *Avicranium renestoi* combines this shape with a completely edentulous rostrum and palate (figure 3*e*). The construction of the orbit differs from that in most Triassic diapsids, in which the cavity is directed anterolaterally [33,41,42]. In *Av. renestoi*, the frontal, postfrontal and postorbital all contribute to a transversely broad postorbital septum, which directs the orbital cavity anteriorly (figure 3*c*). The analogous postorbital process in maniraptorans integrates processes of the frontal, squamosal and laterosphenoid [43]. In most birds, the process is formed primarily by a cartilaginous expansion of the laterosphenoid. Renesto & Dalla Vecchia [40] also suggested binocular vision for *Me. preonensis*, based on the tapering rostrum and broadened orbital and temporal regions.

The endocranium preserves some of the most striking departures of the *Av. renestoi* from other Triassic reptiles. The contribution to the braincase of the paired frontal and parietal bones is both broad transversely and tall dorsoventrally. This contribution is so prominent that the contributing portion of the frontal is domed dorsally well above the orbital margin (figure 3*a*). This corroborates the hypothesis by [40] that the Italian drepanosaurid *Me. preonensis* had an inflated, ‘bulging’ skull roof [39, p. 251]. Among diapsid reptiles, a similar shape otherwise only occurs in maniraptorans [44–47] and some pterosaurs [48,49], taxa that possess enlarged brains relative to other Mesozoic diapsid groups. The reconstructed dorsal surface of the endocast of the *Av. renestoi* resembles those of Pterosauria and maniraptorans in that the cerebrum is large and broad, occupying much of the anteroventral length of the frontal [49–51]. An additional large lobe is formed by the posterior portion of the parietal, likely the optic lobes (based on comparisons with *Alligator mississippiensis* and *Gallus domesticus* in [52]). The anterior outlet of the osseous braincase in *Av. renestoi* is also transversely broad; the prootics angle strongly medially at their anterior tips to meet dorsolaterally inclined clinoid processes of the parabasisphenoid. A brain enlarged in the way this endocast suggests is otherwise unknown in a Triassic reptile (figure 3*a*). Past studies correlate the enlargement of the brain in pterosaurs and maniraptorans with the adaptation of those taxa to flight—it may be that the enlargement of the drepanosaurid brain followed a similar path to an adaptation to the three-dimensional environments required by arboreality, precision grasping and enhanced stereoscopic vision [29,40,49,50,53]. The inclination of the occipital condyle relative to the long axis of the skull is unclear, owing to the disarticulation of the *Av. renestoi* holotype. In our reconstruction, the occipital condyle is slightly posteroventrally inclined relative to the long axis of the skull, in contrast to the strong posteroventral inclination described for *Me. preonensis* [40]. However, the distortion of the skull of *Av. renestoi* may obscure the original shape of the craniocervical articulation.

### 3.2. Plesiomorphic traits

The anatomy of the suspensorium and other morphologies of the braincase stand in stark contrast to the ‘advanced’ features of the skull roof and rostrum. The squamosal is a dorsoventrally tall, anteroposteriorly broad bone. It exhibits both lateral and posterior laminae that frame the quadrate on those sides, as in archaic eureptiles (e.g. *Captorhinus aguti* [43,54]) and diapsids (e.g. *Araeoscelis gracilis* [55]). This contrasts with the condition in younginiform and saurian reptiles (appendix C), in which the quadrate is only framed laterally. In younginiform and saurian taxa, the quadrate also extends dorsally to fit into a fossa on the ventral surface of the squamosal—a feature absent in *Av. renestoi*. The quadrate itself is dorsoventrally short and vertically oriented, lacking the posterior embayment in most early saurian reptiles (figure 3*f*) [33,56–58].

The braincase exhibits a number of traits more commonly found in non-saurian diapsids. The occipital condyle exhibits a deep, posterior depression (=notochordal pit) across much of its surface and the basal tubera barely extend ventrally below the condyle (similar to *Ca. aguti* [59], *Ar. gracilis* [55]) (figure 3*d*). The foramen ovale is extremely large and extends to the ventralmost margin of the braincase. The stapes is massive, with a footplate that entirely fills the foramen and a lateral stem that is larger in all dimensions than the paroccipital process of the opisthotic (figure 3*d*). Foramina ovale and stapedes of this great size are common in early amniotes [59,60], but they are substantially smaller in younginiform diapsids (e.g. *Youngina capensis* [61]) and early saurians (e.g. *Mesosuchus browni* [60], *Prolacerta broomi* [58]). There is no evidence of a laterosphenoid ossification, as in Archosauriformes [62–64].

The plesiomorphic diapsid characters of the skull in *Av. renestoi* strongly suggest a plesiomorphic ear. Extant reptiles possess a tympanic membrane framed anteriorly by the concavity of the quadrate, which medially contacts a cartilaginous extracollumella, which in turn meets a very slender, osseous stapes [65,66]. The absence of an embayed quadrate and tympanic crest in *Av. renestoi* suggests the absence of a tympanic membrane. The large foramen ovale with prominent contributions by parabasisphenoid and basioccipital is more common in non-younginiform and non-saurian amniotes, as is the large stapes [59,65,66]. Thus, *Av. renestoi* lacks the major osteological correlates of impedance-matched hearing. An atympanic condition occurs in a number of extant lepidosaurs (e.g. chameleons, *Sphenodon*), although these taxa exhibit a slender stapes and a condyle–cotyle articulation between quadrate and squamosal and are widely considered to have undergone secondary loss of external ears. The archaic ear morphology in *Av. renestoi*, in concert with the other plesiomorphic amniote traits discussed above, contrasts sharply with the comparatively ‘advanced’ condition in most Triassic Sauria [58,60,67].

## 4. Phylogenetic analysis

**Figure 4.**
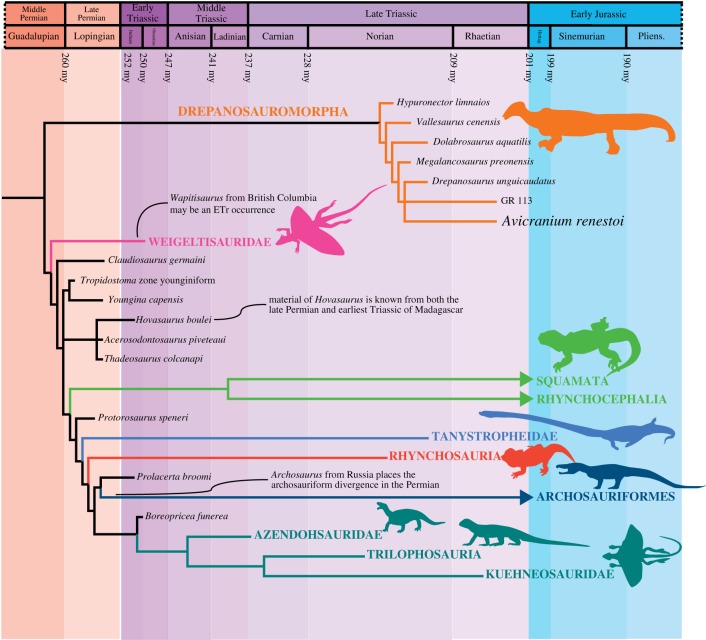
Strict consensus of most-parsimonious trees based on the phylogenetic analysis presented herein. *Petrolacosaurus kansensis* (not shown) was designated as the outgroup. Taxa listed in all-capitals are represented by multiple species-level terminal taxa in the analysis. The complete species-level topology is presented in figures 8 and 9.

In the light of the extensive new data on the cranial anatomy of Drepanosauromorpha provided by AMNH FARB 30834, we integrated the taxon into a phylogenetic analysis focused on terrestrial Permo-Triassic Diapsida and early Sauria (modified from [10,11,68]). We present analysis parameters and detailed results in appendix C. In the most-parsimonious trees, Drepanosauromorpha is recovered as an extremely early-diverging clade of Diapsida, occurring outside of a clade including Permian ‘younginiform’ diapsids and Sauria (figure 4). The oldest-known younginiform diapsid (herein referred to as *Tropidostoma* Zone *Youngina*) dates to the lowermost Upper Permian [69], suggesting that the lineage including drepanosauromorphs must have originated by the end of the Middle Permian (approx. 260 Myr). This phylogeny also recovers Kuehneosauridae, typically found as the sister taxon of Lepidosauria in cladistic analyses of early Diapsida (e.g. [70–72]), as deeply nested within Archosauromorpha (postulated in [73]).

## 5. Discussion and conclusion

These results indicate that drepanosauromorphs represent a deep divergence within Diapsida, earlier than that of crown-group reptiles, but one that persisted through the PTE and radiated deep within the Triassic [11,27]. A number of the non-saurian diapsids included in this analysis are taxa that also survived the PTE (Weigeltisauridae per [74], *Hovasaurus boulei* per [75]), indicating that the survival of drepanosauromorphs among non-crown-group reptiles was not a unique event.

Our revised phylogeny, combined with the extensive character data provided by the *Av. renestoi* holotype, strongly supports the hypothesis that Drepanosauromorpha are non-saurian diapsids. Phylogenetic analyses have long recognized that a number of crown-group reptile lineages (mostly early archosauromorphs) had diverged by the PTE, despite their initial appearance in the fossil record in the Triassic Period [9,10,33,63]. We tested hypothetical placements of drepanosauromorphs among crown-group reptiles through constraint analyses, but found these to be substantially less parsimonious (appendix C). That result, along with the recognition of numerous other non-crown-group lineages within the Triassic indicates that the Triassic diapsid radiation was far more phylogenetically heterogeneous than traditionally realized.

The general bird-like shape of the drepanosaurid rostrum has long been recognized, owing to complete but crushed specimens from the Upper Triassic of Italy. The three-dimensional preservation of AMNH FARB 30834 adds substantially to the bird-like features of the skull, including the frontated orbits and presumed binocular vision, the absence of teeth, possible fusion of the premaxillae and the inflated endocranium. However, these features occur in conjunction with a strikingly plesiomorphic braincase, suspensorium and postcranial skeleton [27]—features that strongly support the hypothesis that these bird-like features are entirely convergent. Bird-like features have been noted in a number of small Triassic diapsids—including *Longisquama insignis* and the putative stem-bird *Protoavis texensis*—which have been used to support the hypothesis that key features of the bird skull evolved very early in the Mesozoic [76,77]. This conception of bird evolution stands at odds with the fossil record of Theropoda, which suggests the gradual acquisition of avian cranial features throughout the Jurassic and Cretaceous [78–80]. The mosaic anatomy of *Av. renestoi* instead supports the hypothesis that several bird-like traits first emerged in a Triassic diapsid lineage entirely outside of crown-group reptiles [36].

The brain of *Av. renestoi* differs greatly from that in most Permian and Triassic diapsids. The cerebrum is substantially wider than the olfactory tracts and the endocranium occupies a substantial proportion of the transverse width of the skull, distinctly similar to the brains of maniraptorans (e.g.[50,81,82]), living birds (e.g.[83–85]) and pterosaurs (e.g. [49,86]). Many authors have suggested that the proportional expansion of brain and cerebrum size in these taxa is an adaptation to the sensory complexity required for navigating three-dimensional habitats [87–89]. The anteriorly directed orbits in *Av*. *renestoi*, coupled with the hypothesized arboreal habitat for drepanosauromorphs [27,39] suggest a complex sensory life for the animal and may explain the similarities in brain shape to flying and arboreal taxa. Further testing of this hypothesis requires better preserved endocasts and reconstruction of the vestibular apparatus of other drepanosauromorphs.

This phylogenetic study, in concert with the bird-like characters of the skull of *Av. renestoi*, increases the known disparity achieved by terrestrial diapsid reptiles during the Triassic Period and extends the pattern of morphological convergence on later Mesozoic lineages during the Triassic beyond Archosauromorpha into a non-crown-group reptile clade. This and similar discoveries demand constant re-evaluation of the phylogenetic diversity and morphological disparity of fossil groups involved in the recovery from the PTE.

## Supplementary Material

Supplemental Appendix A: A. Modifications to characters and codings from the matrix of Nesbitt et al. (2015).; Appendices for Main Text PDF (A–C)

## Figures and Tables

**Figure 5.**
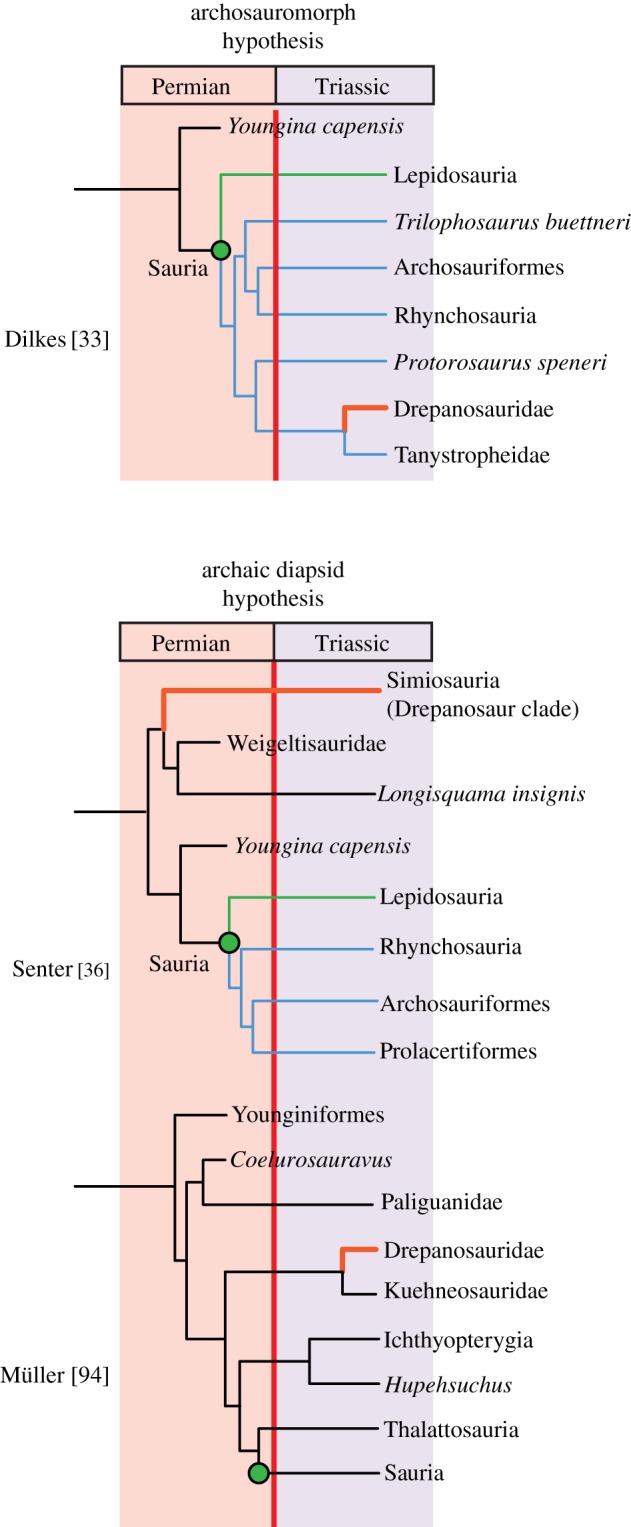
Simplified alternative phylogenetic hypotheses for the affinities of Drepanosauromorpha based on past analyses, presented as stratigraphically calibrated trees.

**Figure 6.**
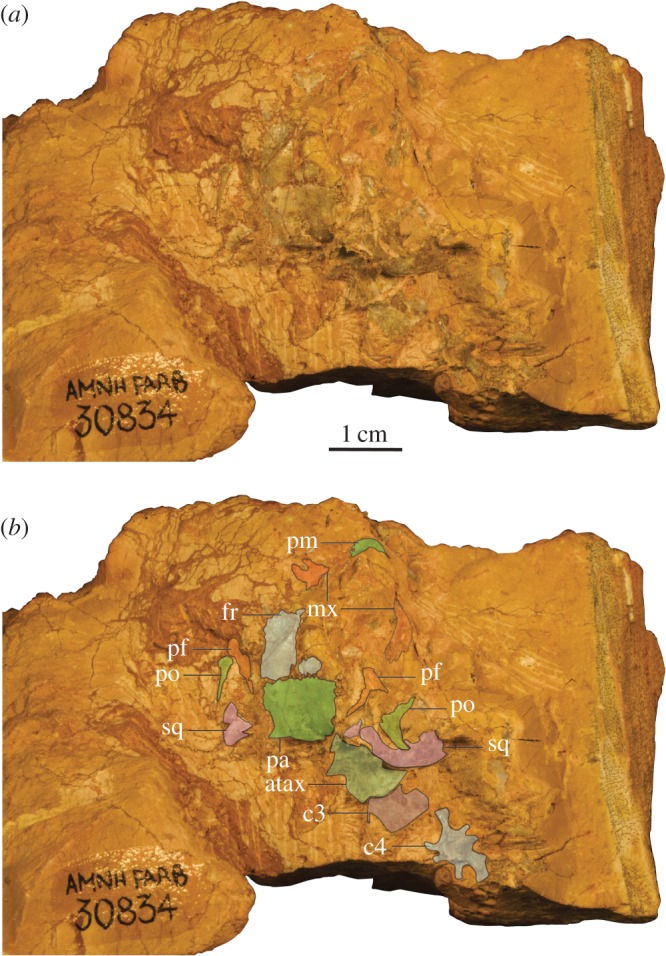
Photograph of the block containing the holotype specimen of *Avicranium renestoi* (AMNH FARB 30834) in dorsal view (*a*) without interpretive callouts and (*b*) with interpretive callouts, indicating the positions of exposed bones. Abbreviations: atax, atlas–axis complex; c3, cervical vertebra 3; c4, cervical vertebra 4; de, dentary; fr, frontal; mx, maxilla; pf, postfrontal; pl, palatine; pm, premaxilla; po, postorbital; sq, squamosal.

**Figure 7.**
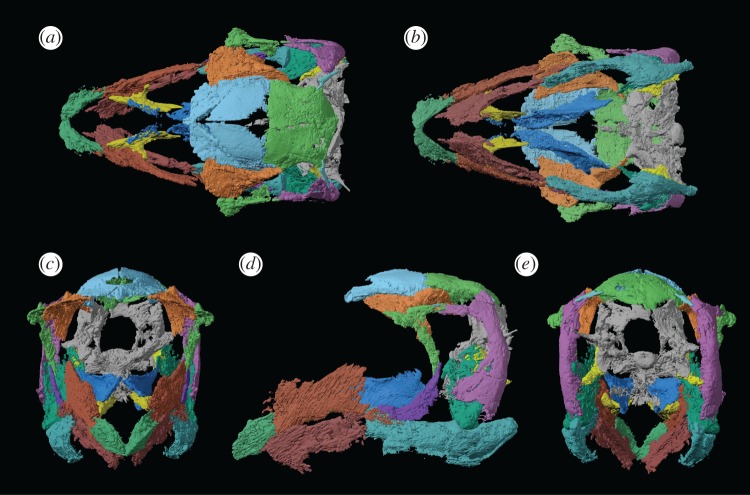
Three-dimensional reconstruction of the skull of *Avicranium renestoi* rendered in Maya 2016 in (*a*) dorsal, (*b*) ventral, (*c*) anterior, (*d*) left lateral and (*e*) posterior views. Elements are colour-coded as in main text (figure 1).

**Figure 8.**
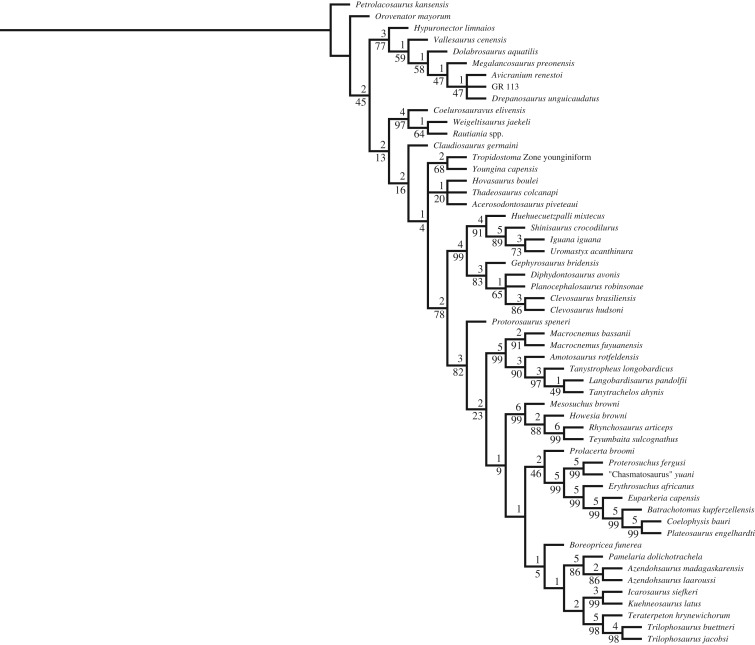
Strict consensus of the primary parsimony analysis presented in this paper. Numbers above the nodes are Bremer values (=decay indices). Numbers below the nodes are frequency differences resulting from the jackknife analysis.

**Figure 9.**
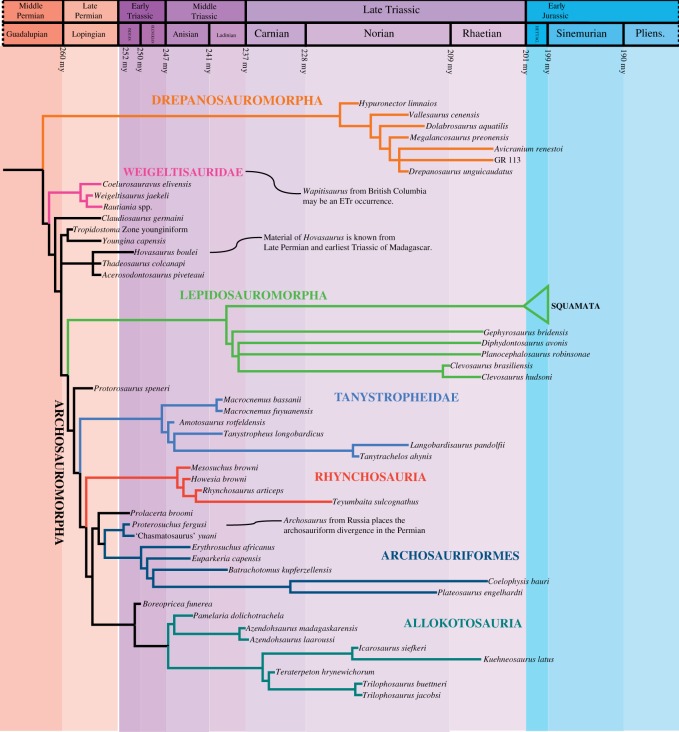
Strict consensus of the primary parsimony analysis stratigraphically calibrated.

**Figure 10.**
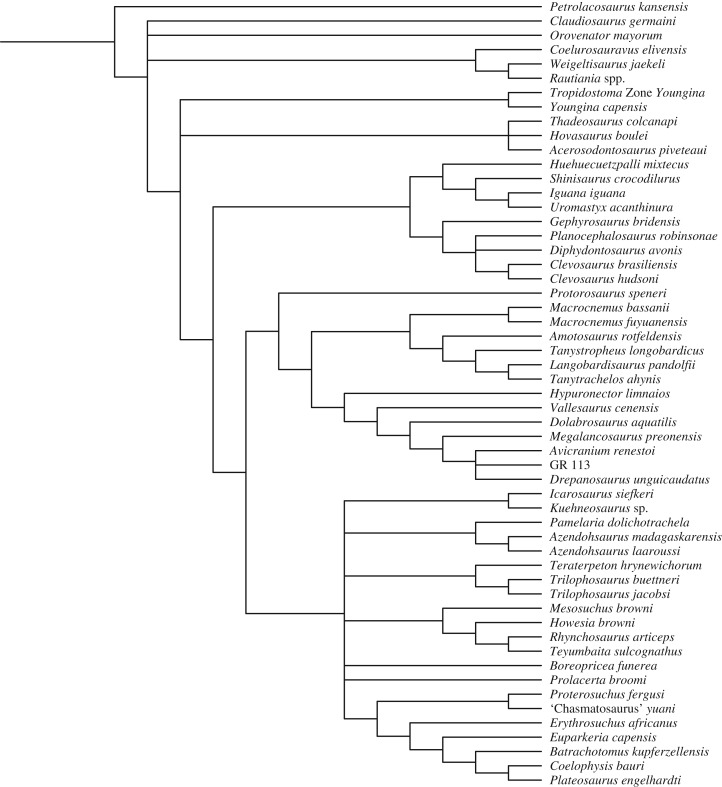
Strict consensus of the analysis constraining Drepanosauromorpha and Tanystropheidae as sister taxa.

**Figure 11.**
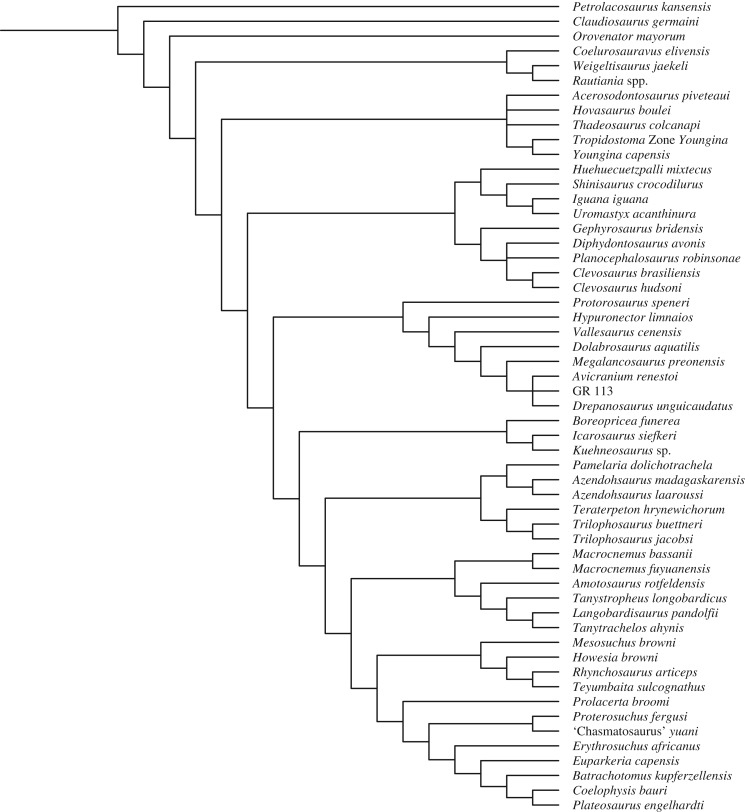
Strict consensus of the analysis constraining Drepanosauromorpha and *Protorosaurus speneri* as sister taxa.

**Figure 12.**
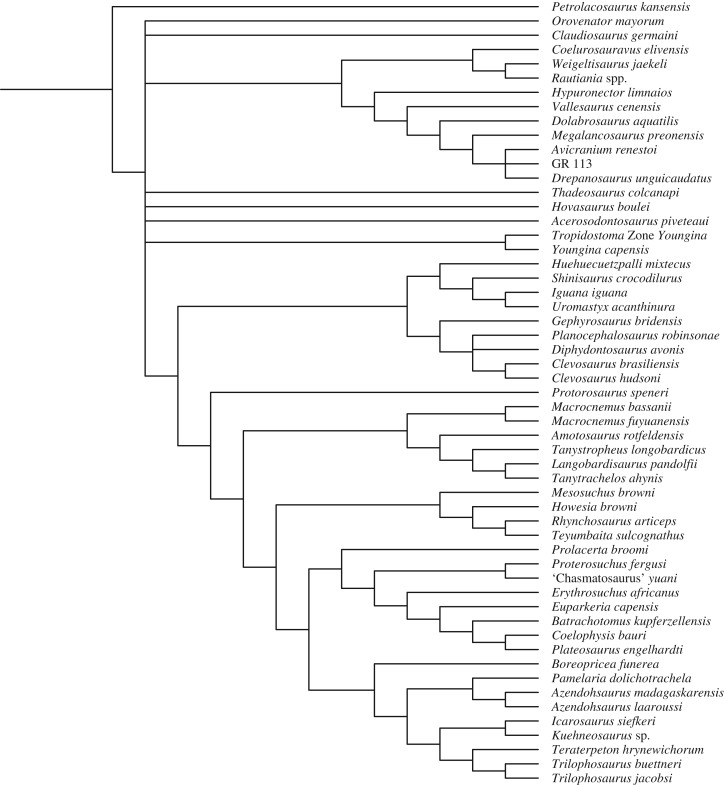
Strict consensus of the analysis constraining Drepanosauromorpha and Weigeltisauridae as sister taxa.

**Figure 13.**
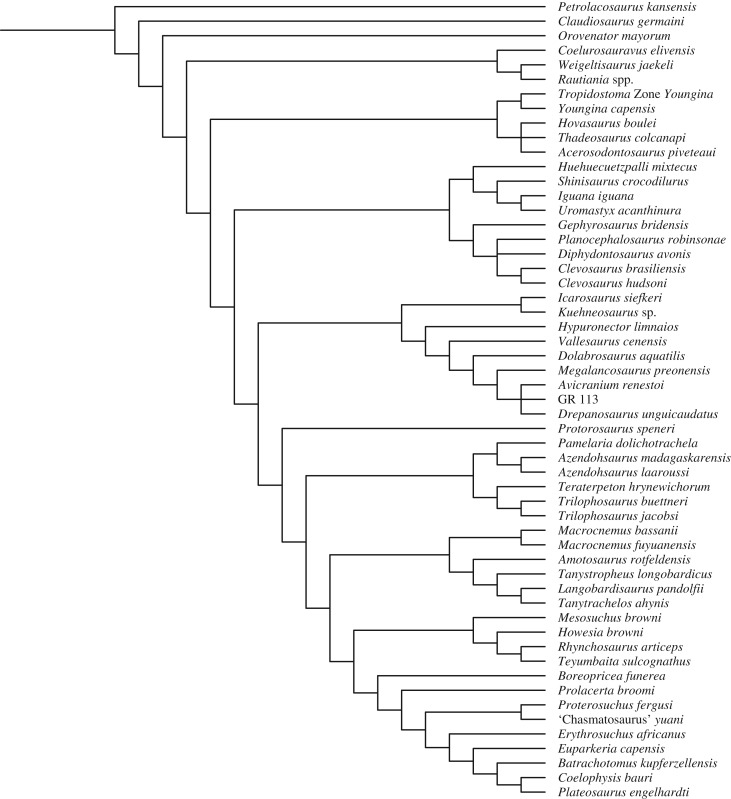
Strict consensus of the analysis constraining Drepanosauromorpha and Kuehneosauridae as sister taxa.

## Data accessibility

The CT datasets that formed the basis of this study are archived on Dryad (http://dx.doi.org/10.5061/dryad.f5q10). The phylogenetic data matrix is available on the Dryad site in TNT format and also on Morphobank (www.morphobank.org) as project 2214. The full list of taxa, characters and phylogenetic methodologies are presented in appendix C.

## Appendix A. Review of past phylogenetic hypotheses for the position of Drepanosauromorpha

In 1980, Giovanni Pinna described the holotype specimen of *D. unguicaudatus* from the Upper Triassic Zorzino Limestone of northern Italy [32]. In that paper and a subsequent description [90], he considered the specimen to be an early lepidosaur, an identification refined to the ‘suborder Eolacertailia’ in later papers [25, p. 1127]. This referral was made based on general morphological comparisons and pre-cladistic diagnoses of Lepidosauria. Berman & Reisz [30] referred the New Mexican reptile *Dolabrosaurus aquatilis* from the Petrified Forest Member of the Chinle Formation to Drepanosauridae, which they also considered to be lepidosaurian. In all the aforementioned cases, the authors perceived a mixture of plesiomorphic and derived diapsid characters in the specimens.

At roughly the same time as the description of the *Drepanosaurus* holotype, Calzavarra *et al.* [91] described a partial skull and skeleton of a small reptile from the Upper Triassic Dolomia di Forni of Italy as the holotype specimen of *Me. preonensis*. They referred to the specimen as a ‘thecodont’ (i.e. early archosauriform). An early phylogenetic study by Evans [73] suggested that *Megalancosaurus* was closer to ‘prolacertiforms’, an arrangement of long-necked early archosauromorphs including *Protorosaurus* and *Tanystropheus*. It would not be until the mid-1990s, when some specimens referred to *Drepanosaurus* were correctly recognized as postcranial skeletons of *Megalancosaurus*, that a close relationship between the putative lepidosaur and ‘thecodont’ was recognized [26]. Renesto [26] also hypothesized that *Megalancosaurus* was closely related to ‘prolacertiforms’.

In 1993, Feduccia and Wild published the hypothesis that *Megalancosaurus* was not only an early-diverging archosaur but also a close relative of birds [77]. They noted a number of cranial features (e.g. a bird-like beak with small teeth; large, bird-like orbit; enlarged braincase) and postcranial features (e.g. slender, strap-like scapula; putative fused clavicles) supporting their hypothesis. This hypothesis was not presented in a rigorous context with a phylogenetic analysis, and the only subsequent study to include both birds and drepanosaurs [36] did not resolve a close relationship between the two.

Systematists have performed numerous cladistics analyses of early diapsid and archosauromorph relationships incorporating a sample of drepanosaurs. In his unpublished PhD thesis analysis, Merck [92] included both *Drepanosaurus* and *Megalancosaurus* into a large-scale study of Permo-Triassic Diapsida. He recovered the two in a clade of Archosauromorpha that was sister to a marine reptile clade including Thalattosauria, Sauropterygia and Ichthyopterygia. By contrast, Merck [35] later presented the hypothesis that drepanosauromorphs and weigeltisaurids formed a clade outside of crown-group reptiles. Dilkes [33] corroborated the hypotheses of Evans [73] (for *Megalancosaurus*) and Renesto [26], by recovering a *Drepanosaurus* *+* *Megalancosaurus* clade as sister to Tanystropheidae within Protorosauria. Numerous authors have modified the Dilkes analysis (e.g. [34,41,93], consistently recovering drepanosauromorphs as nested within Archosauromorpha). Renesto *et al.* [27] incorporated the other named drepanosauromorphs into a modified Dilkes [33] analysis, which likewise supported the early archosauromorph position.

By contrast, other analyses have recovered drepanosauromorphs in various positions outside of Sauria altogether, placing them as the sister taxon to either the gliding Weigeltisauridae [35,36] or Kuehneosauridae [94]. In his analysis, Senter [36] recovered drepanosauromorphs outside of Sauria in a clade including Weigeltisauridae and the poorly known Middle Triassic diapsid *Lo. insignis*. He dubbed this clade Avicephalia. Renesto & Binelli [28] critiqued the Senter [36] analysis and reanalysed the matrix. Renesto & Binelli [28] incorporated the pterosaur *Eudimorphodon* into their analysis, which nested as the sister taxon to drepanosauromorphs within Avicephalia. However, after correcting some errors in the original Senter [36] matrix, the *Eudimorphodon* + Simiosauria clade was recovered as the sister taxon to Archosauriformes. Renesto *et al.* [27] made brief reference to the possibility of a close relationship between pterosaurs and drepanosauromorphs.

In a later iteration of the Müller analysis, Bickelmann *et al.* [95] noted that drepanosaurs acted as a wildcard taxon following the addition of new operational taxonomic units. This phylogenetic instability was attributed to the meager amount of character data coded for drepanosaurs in most analyses, owing to the crushing distortion in nearly all drepanosaur skeletons (e.g. Renesto *et al.* [27]). A summation of the cladistics hypotheses for the affinities of drepanosauromorphs is presented in appendix A and figure 5.

The absence of a coherent hypothesis for the relationships of this group has implications for interpreting the extreme ecomorphology of drepanosauromorphs and the Permo-Triassic radiation of diapsid reptiles. The hypotheses by Dilkes [33] and Renesto [26] suggest that drepanosaurs are deeply nested among crown-group reptiles within the early archosauromorph radiation, specifically within a clade of long-necked, small-headed ‘protorosaurs’. By contrast, the hypotheses of Merck, Senter and Müller suggest that drepanosaurs are not crown-group reptiles, but instead are closely related to archaic Palaeozoic lineages. The members of these lineages are typically smaller in body size, and both hypotheses suggest that the sister taxon of drepanosaurs were extreme gliding specialists. Resolving the ancestry of drepanosauromorphs provides important context for the diversification of small diapsids in the Permian and Triassic.

## Appendix B. Parameters of micro-computed tomography scan, digital segmentation, three-dimensional visualization and rendering of skull bones

The state of preparation of AMNH FARB 30834 and the elements exposed on the surface of the block can be seen in the photographs in figure 6. AMNH FARB 30834 was µCT scanned at the Duke University Shared Material Instrumentation Facility (Durham, NC, USA) by technician James Thostenson. The specimen was scanned at a resolution of 0.0448 mm for a total of 1998 slices (190 kV, 78 mA). The contrast between the bone and matrix is somewhat problematic. Many of the bones contain dense, radiopaque material that appears bright white on the scan slices. These are surrounded by diffuse grey halos. The CT data (in DICOM format) are available for download on Data Dryad under the title ‘Data from: A bird-like skull in a Triassic diapsid reptile increases heterogeneity of the morphological and phylogenetic radiation of Diapsida’.

The specimen was digitally segmented in VG Studio Max 2.2. Based on the visible bone of the frontal and parietals on the dorsal surface of the black, the grey halos are reflective of the true extent of the bone. As such, clusters of the white material were first segmented. These were then expanded to incorporate the diffuse halos. Figure 1 was rendered in VG Studio Max, using the ‘Volume Render (scatter)’ option. We then extracted individual bones as STL surfaces.

The model of the rearticulated drepanosaurid skull was constructed using Maya (v. 2016, Autodesk). Disarticulated bones were fitted together using contact surfaces visible in the extracted surface files. Multiple angles on the rearticulated skulls are presented in figure 7. The endocast in figure 3*a* was reconstructed as a three-dimensional surface using the ventral surfaces of the right frontal and parietal in AMNH FARB 30834. The surface was mirrored and imaged in Maya 2016.

## Appendix C. Phylogenetic analysis: data matrix and results

The phylogenetic dataset used here is a combination of Pritchard *et al.* [68] (and its expansion in Nesbitt *et al.* [10]) and Pritchard *et al.* [11].

**C.1. Museum abbreviations used in comparative description/phylogenetic analysis**

AMNH—American Museum of Natural History (New York, NY, USA)
BP—Evolutionary Studies Institute, University of Witwatersrand (Johannesburg, South Africa)
CM—Carnegie Museum of Natural History (Pittsburgh, PA, USA)
GMPKU—Geological Museum of Peking University (Beijing, China)
GR—Ruth Hall Museum of Paleontology (Abiquiu, NM, USA)
IVPP—Institute of Vertebrate Paleontology and Paleoanthropology (Beijing, China).
MCSN—Museo Civico di Storia Naturali Milano (Milano, Italy)
MCSNB—Museo Civico di Scienze Naturali Enrico Caffi (Bergamo, Italy)
MCZ—Museum of Comparative Zoology (Cambridge, MA, USA)
MFSN—Museo Friulano di Storia Naturale (Udine, Italy)
MNHN—Muséum National d'Histoire Naturelle (Paris, France)
MPUM—Museo di Paleontologia Università di Milano (Milano, Italy)
NHMUK—Natural History Museum of the United Kingdom (London, UK)
NMMNH—New Mexico Museum of Natural History (Albuquerque, NM, USA)
NMQR—National Museum Bloemfontein (Bloemfontein, South Africa).
PIMUZ—Paleontological Institut und Museum (Zürich, Switzerland)
PIN—Paleontological Institute (Moscow, Russia)
SAM—Iziko Museum (Cape Town, South Africa)
SMNS—Staaliches Museum für Naturkunde Stuttgart (Stuttgart, Germany)
TMM—Texas Memorial Museum (Austin, TX, USA)
UA—Université d'Antannanarivo (Antannanarivo, Madagascar)
USNM—United States National Museum of Natural History (Washington, DC, USA)
WMsN—Westfäliches Museum für Naturkunde, Münster (Münster, Germany)
YPM—Yale Peabody Museum of Natural History (New Haven, CT, USA)
ZPAL—Institute of Paleobiology, University of Warsaw (Warsaw, Poland)

**C.2. Taxon list for phylogenetic analyses. Bibliographic references and institutional accession numbers of specimens that were scored based on first-hand examination**

*Acerosodontosaurus piveteaui* [95,96].
*Amotosaurus rotfeldensis*: SMNS 50691, partial skull and anterior portion of skeleton; 53783, multiple associated skeletons; 54784, two skulls, one with associated neck; 54810, dissociated skeleton; 90600, sacrum and partial tail; 90601, articulated maxilla and jugal [97].
*Azendohsaurus laaroussii*: collections of maxillae and dentaries on hand at MNHN. A single premaxilla (MNHN ALM 365-16).
*Azendohsaurus madagaskarensis*: hundreds of specimens accessioned with Université d'Antannanarivo and the Field Museum of Natural History [10,67].
*Batrachotomus kupferzellensis*: cranial and postcranial elements at SMNS [98–100].
*Boreopricea funerea*: PIN 3708/1, 3708/2 [101].
*Chanaresuchus bonapartei*: MCZ 4039 [102,103].
*‘Chasmatosaurus’ yuani*: IVPP V4067, casts of IVPP V36315 [104,105].
*Claudiosaurus germaini*: MNHN MAP 1; SAM K8263, K8266 [106,107].
*Clevosaurus brasiliensis* [108,109].
*Clevosaurus hudsoni* [110]
*Coelophysis bauri* [64,111].
*Dolabrosaurus aquatilis*: CM 28589 [30].
*Drepanosaurus unguicaudatus*: MCSNB 5728 [11,27].
*Euparkeria capensis*: SAM PK 7696 [112,113].
*Erythrosuchus africanus*: NHMUK R3592, NMQR 3765 [114–116].
*Gephyrosaurus bridensis* [117–119].
GR 113: includes all drepanosaurid material preserved on a small block of matrix from the Coelophysis Quarry. Initially described by Harris & Downs [120].
*Hovasaurus boulei*: MNHN MAP 336 [107,121].
*Howesia browni*: SAM PK-5884, 5885, 5886 [122].
*Hypuronector limnaios*: AMNH FARB 1721, 7759 [24].
*Icarosaurus siefkeri*: AMNH FARB 2101 [123].
*Kuehneosaurus latus*: AMNH FARB 7761–7798, NHMUK R 6001, 6002, 6059, 6060 [124,125].
*Langobardisaurus pandolfii*: MCSNB 2883, 4860; MFSN 1921 [126].
*Macrocnemus bassanii*: MCSN BES SC 111, V 457; PIMUZ T.2472, 2477, 4355, 4822 [127,128].
*Macrocnemus fuyuanensis*: GMPKU P-3001 [129].
*Megalancosaurus preonensis*: MFSN 1721; MPUM 6008, 8437 [27].
*Mesosuchus browni*: SAM PK-5882, 6046, 6536, 7416, 7701 [33].
*Pamelaria dolichotrachela* [63,130].
*Petrolacosaurus kansensis*: CM 29904 [131].
*Plateosaurus engelhardti* [42,64,132]
*Proterosuchus* spp. (containing the South African proterosuchid species): BP/1 3393, 4601; NMQR 880, 1484; SAM PK10603 [133,134].
*Protorosaurus speneri*: USNM 442453; SMNS cast of WMsN P 47361 [34].
*Rhynchosaurus articeps* [135,136].
*Shinisaurus crocodilurus* [137,138]..
*Sphenodon punctatum*: YPM R 10646 [139–145].
*Tanystropheus longobardicus*: MCSN BES SC 265, 1018, V 3730; PIMUZ T/1277, T/2819 [146,147].
*Tanytrachelos ahynis*: AMNH FARB 7206; VMNH 2826, 3423, 120015, 120016, 120019, 120042, 120043, 120046, 120047, 120048, 120049; YPM VP 7482, 8600 [68,148].
*Teraterpeton hrynewichorum* [93].
*Teyumbaita sulcognathus* [149,150].
*Thadeosaurus colcanapi*: MNHN MAP 360 [106,151].
*Trilophosaurus buettneri*: several dozen specimens in the collections of TMM, primarily TMM 31025-140 [10,152,153].
*Trilophosaurus jacobsi*: several dozen specimens in the collections of NMMNH (for skull data, primarily NMMNH P-41400) [154].
Tropidostoma Zone younginiform: SAM PK 7710, 8565, 10818 [69].
*Uromastyx acanthinura*: YPM R 13525.
*Vallesaurus cenensis*: MCSNB 4751 [27].
*Youngina capensis*: AMNH FARB 5561; BP/1 375, 2871, 3859; SAM PK 7578, 10777 [58,61,121].

**C.3. Characters for phylogenetic analysis**

The phylogenetic dataset used here is a combination of Pritchard *et al.* [68] (and its expansion in Nesbitt *et al.* [10]) and Pritchard *et al.* [11]. The numbering of characters follows that in Nesbitt *et al.* [10], unless otherwise noted. Bolded characters have been modified in a substantial way from their original use in Pritchard *et al.* [68] and/or Nesbitt *et al.* [10]. Bolded and italicized characters are replacements for characters removed from the dataset in Nesbitt *et al.* [10]. Notes may be found below such characters regarding the grounds for removal. For new or modified characters, references to past or similar usages in other datasets are referenced.

1) Premaxilla external sculpturing: (0) surface is smoothly sculptured, (1) premaxilla is marked by anteroventral striations.

2) Premaxilla, ventral margin, orientation relative to long axis of skull: (0) margin horizontal, roughly inline with maxillary ventral margin; (1) slight downturn, such that the margin trends anteroventrally; (2) extensive downturn, premaxilla extends to ventral margin of dentary. ORDERED.

**3) Premaxilla, anterodorsal process (=nasal process): (0) present, separating the nares; (1) absent or reduced.**

— Modification of Character 3 of Pritchard *et al.* [68] and Nesbitt *et al.* [10] after recognizing taxa with internarial bars and a reduced anterodorsal process of the premaxilla (see Character 252).

4) Premaxilla, posterodorsal process (=maxillary process, subnarial process): (0) absent, such that premaxilla contributes a small ventral margin for the external naris; (1) posterodorsal process present, framing the posteroventral margin of the external naris.

5) Premaxilla, posterodorsal process (=maxillary process, subnarial process), length: (0) short, failing to exclude maxilla from narial margin; (1) long, excluding maxilla from narial margin; (2) extremely long, reaching the anteriormost part of the prefrontal.

6) Premaxilla, posterodorsal process, maxilla contact: (0) simple, straight suture; (1) margin/knob on the posterior margin of the posterodorsal process of the premaxilla fits into notch in the anterior surface of the maxilla.

7) Maxilla, ventral margin, shape: (0) horizontal, (1) convex.

8) Maxilla, posterolateral surface: (0) directly adjacent to alveolar margin, (1) lateral process of maxilla present, creating distinct space between maxillary alveoli and posterolateral surface of the maxilla.

9) Nasal, contact with prefrontal, orientation: parasagittal, (1) oriented anterolateral.

***10) Maxilla, lateral surface near anteroposterior midpoint: (0) marked by subequal neurovascular foramina, (1) bears single neurovascular foramen that is anteroposteriorly longer than all others.***

— This character replaces Character 10 of Nesbitt *et al.* [10], which described the presence of a midline contact of the prefrontal bones. This state is known only in Choristodera, which are not represented in this dataset. It has thus been removed.
— The novel character here describes an enlarged neurovascular opening in the maxillae of *Trilophosaurus* and *Teraterpeton*.

11) Lacrimal, facial contribution: (0) forms a portion of lateral surface of the face that reaches anteriorly to the external naris; (1) forms a portion of the lateral surface of the face but does not reach external naris; (2) limited to orbital margin. ORDERED.

12) Lacrimal, facial contribution, dorsal portion: (0) extends dorsally to reach the ventral margin of the nasal; (1) externally, lacrimal fails to reach nasal.

13) Antorbital fenestra: (0) absent, (1) present.

14) Frontal, fusion to contralateral frontal: (0) unfused, suture patent; (1) fused in the midline, no clear suture dorsally.

15) Frontals, shape: (0) maintains transverse width throughout its anteroposterior length; (1) gradual transverse expansion towards posterior margin of bone; (2) abrupt transverse expansion in postorbital region; (3) tapered posteriorly due to transverse breadth of postfrontals.

16) Frontal, shape of contact with parietal: (0) roughly transverse in orientation; (1) anteriorly convex, U-shaped contact, with frontal exhibiting posterolateral processes at contact.

17) Frontal and postfrontal, dorsal surfaces, texture: (0) relatively smooth; (1) distinct pitting.

18) Postfrontal: (0) present, (1) absent as discrete ossification.

19) Parietal, fusion to contralateral parietal: (0) unfused to one another, patent suture; (1) fused at the midline, no distinct suture.

20) Parietal, dorsal surface: (0) flattened skull table; (1) dorsal exposure of parietal forms a raised margin elevated above lateral excavation for jaw adductor musculature; (2) thin blade like sagittal crest. ORDERED.

21) Parietal, posterolateral (=post-temporal) processes, orientation: (0) roughly transverse, (1) angled strongly posterolaterally.

22) Pineal foramen: (0) present, (1) absent.

23) Pineal foramen, position on skull roof: (0) entirely surrounded by parietals, (1) situated within the frontoparietal suture.

24) Postparietals: (0) absent as discrete ossifications; (1) present.

25) Postparietal, fusion in midline: (0) unfused, with patent suture evident; (1) fused as a midline interparietal.

26) Postorbital, medial process: (0) absent, with contributions of the frontal, parietal, and/or postfrontal forming the posterodorsal orbital margin; (1) present, postorbital contributing to posterodorsal orbital margin.

***27) Postorbital, medial contact with frontal and parietal: (0) present, (1) absent with postfrontal fitted in between.***

— This character replaces Character 27 of Pritchard *et al.* [68] and Nesbitt *et al.* [10], which described the relative position of the medial process of the postorbital to the postfrontal. We have incorporated this character to distinguish taxa that truly lack a contact between the postorbital and the midline skull roof elements. Character 254 also partly addresses this morphology, describing the shape and anteroposterior dimensions of the dorsal exposure of the postfrontal.

28) Postorbital, posterior process, anteroposterior length: (0) contributes to lateral margin of supratemporal bar, but does not reach the posterior aspect of the infratemporal fenestra; (1) contributes to the entire anteroposterior length of the supratemporal bar reaching the posterior aspect of the infratemporal fenestra.

29) Infratemporal fenestrae, conformation: (0) present as distinct opening, framed by squamosal, postorbital and jugal; (1) postorbital, jugal and squamosal fit against one another as a lateral temporal plate.

30) Jugal, lateral surface, ornamentation: (0) unornamented; (1) distinct anteroposteriorly trending shelf present.

31) Jugal, dorsal process, contact with squamosal: (0) absent; (1) present.

32) Jugal, posterior process: (0) absent; (1) present but failing to contact the quadratojugal posteriorly; (2) present, contacting the quadratojugal posteriorly. ORDERED.

**33) Squamosal, lateral (=descending) process/flange: (0) anteroposteriorly broad, covering the quadrate entirely in lateral view; (1) anteroposteriorly slender, partially exposing quadrate; (2) absent. ORDERED.**

— Pritchard *et al.* [68] and Nesbitt *et al.* [10] described the presence and absence of the lateral/descending process of the squamosal in their Character 33 and the relative anteroposterior breadth of that process in Character 34. Here, we present a composite of those characters, with a slender lamina seen as an intermediate between the absence of the structure and the anteroposteriorly broad laminae of early diapsids.

***34) Squamosal, posterior lamina: (0) present, covering much of posterior aspect of quadrate; (1) absent, posterior aspect of quadrate exposed in occipital view.***

***35) Squamosal, contact with quadrate: (0) braces quadrate laterally; (1) dorsal portion of bone forms broad contact with dorsal surface of quadrate.***

36) Supratemporal: (0) absent as discrete ossification, (1) present.

37) Tabulars: (0) absent, (1) present.

38) Quadratojugal: (0) present, (1) absent as distinct ossification.

***39) Quadratojugal, anterior process: (0) present, (1) absent.***

— Neither Pritchard *et al.* [68] nor Nesbitt *et al.* [10] include a character describing the presence or absence of an anterior process of the quadratojugal. This choice was made in the light of the apparent lack of taxa bearing both a complete lower temporal bar and the absence of an anterior process of the quadratojugal, such that coding for this process and the lower temporal bar would be redundant. However, Ezcurra & Butler [134] note the presence of complete lower temporal bars in *Proterosuchus* specimens lacking quadratojugal anterior processes. As the characters do appear independent, we integrate this character into the present analysis.

**40) Quadratojugal, anterior process, shape: (0) paralleling dorsal and ventral borders, (1) anteriorly tapering anterior process.**

— This character may be found as Character 39 in Pritchard *et al.* [68] and Nesbitt *et al.* [10]. It here replaces the original Character 40 in those datasets, which described the relative dorsoventral height of the quadratojugal. However, first-hand study of certain taxa with supposedly dorsoventrally low quadratojugals (e.g. *Ar. gracilis*, MCZ 4036) suggests that the full dorsoventral height of the bone is difficult to assess due to the anteroposterior breadth of the squamosal. As such, we have removed that character pending further study of this state in early Diapsida.

41) Quadrate, posterior margin, shape: (0) straight, vertically oriented; (1) concave, excavated.

42) Quadrate, lateral flange (=tympanic crest): (0) absent, quadrate has no lateral expansion; (1) present.

***43) Quadrate, tympanic crest, conch: (0) absent, (1) present as deep concavity on posterior surface of crest.***

— We have removed the original Character 40 of Pritchard *et al.* [68] and Nesbitt *et al.* [10], which described the position of the quadrate foramen either between quadratojugal and jugal or within the quadrate. In the taxa studied in this analysis, the only species with a purely quadrate-enclosed foramen are also those that lack a discrete quadratojugal ossification. As such, we have removed the foramen character to avoid redundancy.
— This character describes the development of a deep posterior concavity on the tympanic crest in many Lepidosauria (e.g. *G. bridensis* [117], *U. acanthinura* (YPM R 13525)). Taxa coded as ‘0’ for Character 42 are coded as ‘-’ for this character.

44) Palatal teeth: (0) present, (1) absent.

45) Vomer, teeth: (0) present, (1) absent.

— Taxa coded as ‘1’ for Character 44 are coded as ‘-’ for this character.

46) Vomer, contact with maxilla: (0) absent, vomer only contacts premaxilla; (1) present, vomer premaxilla contact expands onto maxilla.

47) Palatine teeth: (0) present, (1) absent.

— Taxa coded as ‘1’ for Character 44 are coded as ‘-’ for this character.

***48) Palatine. Lateral tooth row, dental morphology: (0) similar to other palatal teeth; (1) enlarged relative to all other palatal teeth, akin to marginal teeth in size and morphology.***

— This character was not present in the datasets of Pritchard *et al.* [68] nor Nesbitt *et al.* [10]. The original Character 48 described the presence of teeth on the anterior process of the pterygoid. It is accounted for in Characters 49 and 50 of this study.
— This character describes the enlarged palatine teeth of Rhynchocephalia.

***49) Pterygoid, anterior process dentition, medial row (row T3 of Welman [155] and Ezcurra [63]): (0) absent, (1) present.***

— Pritchard *et al.* [68] and Nesbitt *et al.* [10] described only the presence/absence (Character 48) and number of tooth fields (Character 49) on the anterior process of the pterygoid. However, Welman [155] and Ezcurra [63] make compelling cases for the homology of rows on specific regions of the process. Our novel formulations of Characters 49 and 50 in this analysis accommodate these hypotheses.

***50) Pterygoid, anterior process dentition, lateral row (row T2 of Welman [155] and Ezcurra [63]): (0) absent, (1) present.***

— See notes for Character 49.

51) Pterygoid, transverse process, dentition: (0) absent, (1) present.

— This character is included in Pritchard *et al.* [68] and Nesbitt *et al.* [10] as Character 50.

52) Pterygoid, transverse process, dentition, number of tooth: (0) multiple rows (1) one row.

— This character is included in Pritchard *et al.* [68] and Nesbitt *et al.* [10] as Character 51.

53) Pterygoid, midline contact with contralateral pterygoid: (0) absent, (1) present, small contact present at anterior tips; (2) present, broad contact throughout anteroposterior length. ORDERED.

— This character is included in Pritchard *et al.* [68] and Nesbitt *et al.* [10] as Character 52.

54) Pterygoid, transverse process, orientation of long axes: (0) lateral, (1) anterolateral.

— This character is included in Pritchard *et al.* [68] and Nesbitt *et al.* [10] as Character 53.
— We have removed Character 54 of the original datasets of Pritchard *et al.* [68] and Nesbitt *et al.* [10], which described the shape of the midline space framed by the contacting pterygoids (anteriorly tapered or anteriorly curved). However, the curved shape appears to be the simple by-product of an anteroposteriorly elongate pterygoid–pterygoid contact (as in Rhynchosauria). As such, we have eliminated that character from this study to avoid redundancy.

55) Supraoccipital, posterior surface: (0) smooth; (1) distinct dorsoventrally running crest in the midline.

56) Supraoccipital, shape: (0) consists of a flattened posterior lamina, (1) pillar like (U-shaped in dorsal view).

57) Opisthotic, ventral ramus, shape: (0) slender process, (1) distinct club-shaped expansion ventrally.

**58) Opisthotic, paroccipital process, contact with suspensorium: (0) absent, ends freely; (1) present.**

— The original formulation of this character presented in Pritchard *et al.* [68] and Nesbitt *et al.* [10] described the contact between the paroccipital process and the squamosal. However, these codings ignored taxa in which the lateral tip of the paroccipital process contacts the suspensorium at the supratemporal or quadrate [156], depending on the relative development of those elements. For the moment, we retain a single character to describe the presence or absence of a paroccipital process–suspensorium contact, hypothesizing that such a contact is homologous across Diapsida.

**59) Exoccipital, contact with dorsal elements of occiput: (0) exoccipitals columnar throughout their dorsoventral height, forming transversely narrow contact with dorsal occiput elements; (1) exoccipitals exhibit dorsomedially inclined processes which do not meet in the midline; (2) exoccipitals meet dorsally over the foramen magnum excluding the supraoccipital from that opening. ORDERED.**

— This character expands on the original Character 59 in Pritchard *et al.* [68] and Nesbitt *et al.* [10], which only described the presence of the dorsomedially inclined processes of the exoccipitals. In those studies, Character 60 described the presence or absence of a supraoccipital contribution to the dorsal margin of the foramen magnum. However, in studying those taxa that lack an exposure of the supraoccipital on the foramen magnum, we have recognized no taxa that lack dorsomedial processes that do not exhibit a supraoccipital exposure. As the incipient presence of these processes appears to be a necessary intermediate condition between the columnar exoccipitals and the complete exclusion of the supraoccipital from the foramen magnum, we elected to combine the original characters into a single, ordered character for this study.

60) Exoccipital, contact on floor of foramen magnum with contralateral exoccipital: (0) absent, basioccipital contributes to floor of foramen magnum; (1) present, excluding basioccipital from floor of the foramen magnum.

— This character was present in Pritchard *et al.* [68] and Nesbitt *et al.* [10] as Character 61.

**61) Exoccipital, fusion with other braincase elements: (0) unfused to other braincase elements, sutures with basioccipital and opisthotic patent; (1) exoccipital fused to opisthotic; (2) exoccipital fused to basioccipital.**

— This character was modified from Character 60 in Pritchard *et al.* [68] and Nesbitt *et al.* [10], which described the presence of fusion between exoccipital and opisthotic. We have noted the presence of numerous taxa that exhibit a distinct fusion between the basioccipital and exoccipital (e.g. CQ drepanosaurid (AMNH FARB 30834), *Pr. broomi* (BP/1 2675), *Czatkowiella harae* [56]), for which we have introduced an additional state.

62) Opisthotic, paroccipital process, morphology: (0) unflattened and tapered, (1) anteroposteriorly flattened distally.

— This character was present in Pritchard *et al.* [68] and Nesbitt *et al.* [10] as Character 62.

***63) Basioccipital, occipital condyle, posterior surface: (0) exhibits elliptical notochordal depression that occupies much of posterior surface of condyle; (1) exhibits narrow ‘pinprick’ notochordal pit within posterior surface; (2) condyle is smoothly convex. ORDERED.***

— We introduce this character here to describe both the presence of a notochordal pit within the occipital condyle and the relative development of that pit. As early Sauria exhibit a range of morphologies, including exceptionally broad pits and extremely transversely narrow pits, we include an intermediate state between an ‘unpitted’ condyle and very broad, prominent pits.

**64) Basioccipital, basal tubera: (0) poorly developed, not extending well ventral of occipital condyle; (1) well developed, extending ventral to level of occipital condyle.**

— The original Character 64 in Pritchard *et al.* [68] and Nesbitt *et al.* [10] described the presence/absence of basioccipital basal tubera. In examining the early sauropsid and diapsid taxa purported to lack such tubera (e.g. *Araeoscelis*, *Captorhinus*), we recognized that the absence of such tubera in these taxa was more accurately described as a weak/incipient development of the tubera in which they did not extend far ventrally relative to the occipital condyle. The character has been rephrased to account for this morphological detail.

65) Parabasiphenoid, cultriform process, dentition: (0) absent, (1) present.

66) Parabasisphenoid, parasphenoid crests: (0) absent, such that there is no ventral floor for the vidian canal; (1) present as prominent ventrolateral extensions of the caudoventral processes, framing the ventromedial floor of the vidian canal.

67) Parabasisphenoid, passage for internal carotid arteries: (0) within lateral wall of braincase; (1) within ventral surface of the parabasisphenoid; (2) passage of the internal carotids does not enter the braincase.

68) Parabasisphenoid, conformation of ventral surface: (0) roughly planar; (1) distinct depression at the suture between the basioccipital and the parabasisphenoid; (2) distinct depression within the parabasisphenoid.

69) Parabasisphenoid, cultriform process: (0) extremely elongate, reaching to the level of the internal nares; (1) shorter, failing to reach internal nares.

70) Parabasisphenoid, basipterygoid process, orientation of long axes: (0) anterolateral; (1) lateral.

71) Parabasisphenoid, abducens foramina: (0) within the dorsum sella; (1) track across dorsal surface of dorsum sella.

72) Laterosphenoid ossification: (0) absent; (1) present, but fails to reach ventral surface of frontals; (2) present reaching ventral surface of frontals. ORDERED.

**73) Prootic, lateral surface, anteroventrally oriented crest (=crista prootica): (0) present, (1) absent.**

— We have added further morphological description than present in Pritchard *et al.* [68] or Nesbitt *et al.* [10] to this character to clarify how we define ‘crista prootica’.

**74) Prootic, anteroventral surface, anterior inferior process: (0) present, framing anterior margin of trigeminal foramen; (1) absent, trigeminal foramen unframed anteriorly.**

— We have added further morphological description than present in Pritchard *et al.* [68] or Nesbitt *et al.* [10] to this character to clarify how we define ‘anterior inferior process’.

75) Prootic, posterolateral surface, contribution to paroccipital process: (0) absent, no contribution to anterior surface of paroccipital process; (1) present, contributes laterally tapering lamina to the anterior surface of the process.

76) Stapes, dorsal process: (0) absent, (1) present.

77) Stapes, foramen for stapedial artery: (0) present, (1) absent.

78) Dentary, anterior portion, symphyseal region of mandible: (0) dentaries do not diverge, (1) tips of dentaries diverge from one another.

79) Coronoid process: (0) absent, (1) present.

80) Surangular, lateral surface, foramen positioned near surangular-dentary contact: (0) absent, (1) present.

81) Surangular, lateral surface, foramen positioned directly anterolateral to glenoid fossa: (0) absent, (1) present.

82) Angular, exposure on lateral mandibular surface: (0) broadly exposed, (1) limited to less than one-third of the dorsoventral height of the mandible.

83) Angular, exposure on lateral mandibular surface: (0) terminates anterior to the glenoid, (1) extends to the glenoid.

84) External mandibular fenestra: (0) absent, (1) present.

85) Splenial, contribution to mandibular symphysis: (0) splenials contribute to symphysis, (1) splenials fail to contribute.

86) Retroarticular process: (0) present as extension of articular and adjacent bones posterior to quadrate articulation, (1) absent.

— Taxa code as ‘1’ for this character are coded as ‘-’ for Character 266.

**87) Articular, fusion to prearticular: (0) absent, (1) present.**

— This character is slightly modified from Character 87 of Pritchard *et al.* [68] and Nesbitt *et al.* [10], which described the fusion of articular and prearticular as a character state describing the ‘composition’ of the retroarticular process. In considering the condition of most Lepidosauria, which bear this fusion, it is more appropriate to simply describe the fusion of these two elements as the character.

88) Marginal dentition on anteriormost portions of premaxilla and dentary: (0) present, (1) absent.

89) Marginal dentition, enlarged caniniform teeth in maxilla: (0) present, (1) absent, maxillary teeth subequal in size.

90) Marginal dentition, serrations: (0) absent, (1) present.

91) Marginal dentition, posterior margin of tooth, shape: (0) convex or straight, (1) concave.

92) Marginal dentition, arrangement on dentigerous surface of maxilla: (0) single row of marginal teeth, (1) multiple zahnreihen in maxilla.

93) Marginal dentition, morphology of crown base: (0) single pointed crown, (1) flattened platform with pointed cusps, (2) mesiodistally arranged cusps.

**94) Marginal dentition, implantation: (0) teeth situated in shallow groove (as in pleurodonty, thecodonty); (1) teeth superficially attached to tooth bearing bones, with limited extension of pulp cavity into the bone teeth; (2) superficially attached to tooth bearing bones, with no extension of pulp cavity into the bone (=true acrodonty).**

— This character has been modified from Character 94 in Pritchard *et al.* [68] and Nesbitt *et al.* [10], incorporating two states for what was regarded as acrodonty (superficial attachment of teeth to dentigerous bones) in the original datasets. The new states describe intermediate stages of acrodonty, as revealed by CT scanning of early rhynchocephalians (described in [157]). In *Planocephalosaurus robinsonae* and *Diphydontosaurus avonis*, teeth appear to be superficially attached to their respective dentigerous bones, but with pulp cavities that extend into said bones. By contrast, in *Sp. punctatum* and *Cle. hudsoni*, the pulp cavities do not invade the dentigerous bones. We order this character under the hypothesis that the reduction in the extent of the pulp cavity as an intermediate condition between rooted teeth and teeth with entirely superficial attachment. Taxa that lack CT investigation of pulp cavity morphology we code as 1 and 2.

95) Marginal dentition, lingual surface: (0) teeth walled by minimal lingual wall, (1) no lingual wall (=pleurodonty).

96) Marginal dentition, lingual surface: (0) teeth walled only by minimal lingual wall, (1) interdental plates are present.

97) Marginal dentition, rooting: (0) tooth crowns are not attached to dentigerous bones teeth, (1) ankylosed to bones of attachment.

98) Marginal dentition, tooth shape at crown base: (0) subcircular, (1) labiolingually compressed, (2) labiolingually wider than mesiodistally long.

***99) Marginal dentition, regionalization: (0) no clear abrupt change from anterior to posterior teeth, (1) clear shift from small anterior juvenile teeth anteriorly to large posterior adult teeth.***

— We removed the original Character 99 from Pritchard *et al.* [68] and Nesbitt *et al.* [10], which described palatal dentition morphology (small, button-shaped teeth versus conical teeth). These poorly defined states require further study, especially of small-bodied taxa on which such characters are difficult to discern (e.g. *Pe. kansensis*, *Y. capensis*). Note that Character 48 describes morphological distinctions in the palatine dentition of Rhynchocephalia.
— This character describes the substantial heterogeneity in tooth size in Rhynchocephalia.

100) Marginal dentition, procumbency: (0) anteriormost marginal teeth have similar apicobasal orientation to posterior teeth, (1) anteriormost teeth are procumbent.

***101) Vertebrae notochordal canal: (0) present, (1) absent.***

— We removed the original Character 101 from Pritchard *et al.* [68] and Nesbitt *et al.* [10], which described the relative concavity of the anterior articular surface of the vertebral centra. No taxa studied first-hand were coded as lacking an anterior concavity to the vertebral centrum; indeed, the only animals coded as such were based on text descriptions of vertebral morphology (e.g. [158] for *Orovenator mayorum*). In the light of the poor definition of this character and its absence in specimens studied first-hand, we elect to remove it from the study.
— This character describes the midline canal in the vertebral centra of many early Diapsida and Lepidosauromorpha (e.g. *G. bridensis* [119], *Cle. hudsoni* [110] and *Sp. punctatum* (YPM R 10646)).

102) Presacral vertebrae, posterior articular surface: (0) planar, (1) concave, (2) convex.

103) Presacral vertebrae, posterior convexity: (0) slight with posteriorly flattened surface; (1) strong, hemispherical.

— Taxa coded as ‘0’ or ‘1’ for Character 102 are coded as ‘-’ for this character.

104) Anterior cervical ribs, shaft, shape: (0) tapering rapidly, roughly triangular in lateral view; (1) ribs taper gradually, elongate and splint like in lateral view.

105) Cervical ribs, anterior process: (0) absent, (1) present.

106) Intercentra in the cervical region: (0) present, (1) absent.

107) Anterior postaxial cervical vertebrae, shape of anterior articular surface: (0) subcircular, roughly equivalent in dorsoventral height and transverse width; (1) compressed with a greater transverse width than dorsoventral height.

108) Cervical vertebrae, ventral keel: (0) present, (1) absent.

109) Anterior postaxial cervical vertebrae, ventral surface, shape excluding keel: (0) convex, rounded; (1) flattened.

110) Cervical vertebrae, costal facet, number: (0) one, (1) two.

111) Anterior postaxial cervical vertebrae, position of diapophysis or dorsal margin of synapophyses: (0) at or near dorsoventral level of pedicles, (1) further ventrally near the dorsoventral midpoint of the centrum.

112) Anterior postaxial cervical vertebrae, costal facets, position relative to one another: (0) distinctly offset from one another, (1) facets very closely appressed to one another with little or no finished bone separation.

113) Anterior postaxial cervical vertebrae, neural spine, shape of base: (0) anteroposteriorly elongate, subequal in length to the neural arch; (1) short spine restricted to posterior half of neural arch.

114) Anterior postaxial cervical vertebrae, neural spine, shape in cross section: (0) transversely narrow, (1) elliptical or circular.

115) Anterior postaxial cervical vertebrae, neural spine, anterior margin, shape: (0) straight and linear; (1) anterodorsal process present, forming an anterior notch.

116) Anterior postaxial cervical vertebrae, neural spine, anterior margin, inclination: (0) posterodorsal, (1) anterodorsal.

117) Anterior postaxial cervical vertebrae, neural spine, dorsal tip: (0) transversely slender, (1) expanded transversely.

118) Mid-cervical vertebrae, neural spine, height: (0) equivalent in height and length to other cervical neural spines; (1) dorsoventrally depressed at anteroposterior midpoints, leaving them little more than midline dorsal ridges.

**119) Cervical vertebrae, postzygapophyses, dorsal surfaces: (0) smooth and rounded, marked by dorsoventrally trall projections (=epipophyses).**

— We have modified the definition of the second state of this character, which initially noted that epipophyses were required to be ‘posteriorly projecting’. In some taxa (e.g. *Az. madagaskarensis* [10], *Pa. dolichotrachela* [130]), there are prominent ridges on the dorsum of the postzygapophyses, but these do not point posteriorly or posterodorsally. As such, we have modified the definition of epipophyses to be more inclusive. Note that Character 271 in this analysis describes the shape and development of these epipophyses.

120) Anterior dorsal vertebrae, position of parapophysis or ventral margin of dorsal portion of synapophysis: (0) partially on lateral margin of centrum, (1) entirely on neural spine.

121) Posterior dorsal vertebrae, position of parapophysis or ventral margin of dorsal synapophysis: (0) partially on lateral margin of centrum, (1) positioned entirely on neural spine.

122) Anterior dorsal vertebrae, pectoral region, number of costal facets: (0) one (=holocephaly), (1) two (=dichocephaly), (2) three (=tricephaly).

123) Posterior dorsal vertebrae, costal facets: (0) single rib facet; (1) inverse L rib facet, suggesting partial confluence of diapophysis and parapophysis; (2) double rib facet.

124) Posterior dorsal vertebrae: (0) ribs fused to costal facets, (1) unfused.

125) Dorsal vertebrae, neural spines, dorsal portion: (0) similar width as the more distal portion of the neural, (1) spine expanded transversely into a flattened tip spine table.

***126) Dorsal vertebrae, neural arches, dorsolateral surfaces: (0) marked by rounded mammillary processes*, (*1*) *smooth*.**

— The original Character 126 in Pritchard *et al.* [68] and Nesbitt *et al.* [10] described the degree of transverse expansion in the dorsal neural spines. We chose to eliminate that character, as it did not account for the nature of that expansion (e.g. whether or not is was formed by a transverse broadening of the bone of the tip of the spine, or if it was formed by mammillary processes just distal to the tip). Further study is definitely needed on vertebral variation in early Diapsida, but for the moment we describe the presence of these processes in the novel character above.

127) Dorsal vertebrae, neural spine, dorsal tip, texturing: (0) marked by pebbly unfinished bone, (1) marked by transverse striations of bone.

— Taxa coded as ‘0’ for Character 125 are coded as ‘-’ for this character.

128) Dorsal vertebrae, intercentra: (0) present, (1) absent.

129) Dorsal vertebrae, neural spines, dorsoventral height: (0) tall, greater in dorsoventral height than anteroposterior length; (1) long and low, lesser in dorsoventral height than anteroposterior length.

130) Dorsal vertebrae, accessory zygosphene zygantrum articulations: (0) absent, (1) present.

131) Second sacral rib, shape: (0) rib is a single unit, (1) rib bifurcates distally into anterior and posterior processes.

132) Second sacral rib, posterior process: (0) terminally blunted, (1) sharp distally.

— Taxa coded as ‘0’ for Character 131 are coded as ‘-’ for this character.

**133) Anterior caudal vertebrae, transverse processes, shape: (0) curve posterolaterally, (1) straight, (2) curved anterolaterally.**

— This character is modified from Character 133 of Pritchard *et al.* [68] and Nesbitt *et al.* [10] in the addition of a third state describing anterolateral curvature of the caudal transverse processes. Examples of this condition occur in *Cla. germaini* (MNHN MAP 1) and *Sp. punctatum* (YPM R 10646).

134) Anterior caudal vertebrae, transverse processes, medial base, orientation: (0) perpendicular to the long axis of the vertebra, (1) angled posterolaterally.

135) Caudal vertebrae, autotomic septa within centra: (0) absent, (1) present.

**136) Chevrons, hemal spine, shape: (0) tapers along its proximodistal length; (1) broadens slightly along its length; (2) broadens distally, forming inverted T-shape broadens distally forming subcircular expansion.**

— Pritchard *et al.* [68] and Nesbitt *et al.* [10] included a state that described chevrons that maintain their anteroposterior breadth along their proximodistal length. This is not strictly the case in most taxa that were coded as such in these analyses; in most early archosauromorphs (e.g. *Protorosaurus speneri*, *Mes. browni*), the chevrons broaden very slightly at their dista ends. The codings have been changed as such.

***137) Chevrons, hemal spine, length: (0) similar in length or shorter than caudal neural spines, (1) substantially longer than caudal neural spines.***

— In Pritchard *et al.* [68] and Nesbitt *et al.* [10], Character 137 described the presence and absence of gastralia. In Nesbitt *et al.* [10], Character 237 introduced a character that described the presence of abundant gastralia or their absence or extremely limited ossification. We elected to eliminate the original iteration of Character 137 for this analysis, as Character 237 is more descriptive.
— We introduce this character to describe the extreme proximodistal elongation of the chevrons in certain early diapsid clades, especially drepanosauromorphs (e.g. *Hy. limnaios* (AMNH FARB 7759), *D. unguicaudatus* (MCSNB 5728)).

***138) Chevrons, hemal spine, curvature: (0) roughly straight, (1) convex anteriorly.***

— Pritchard *et al.* [68] and Nesbitt *et al.* [10] incorporated a character describing the number of pairs of lateral gastralia in each segment. Merck [92] described the presence of two pairs in most non-archosauriform diapsids, and a single pair in Archosauriformes. However, we note that two gastral ossifications are present on each side in coded early archosauromorphs (e.g. *L. pandolfii*, MFSN 1921; *Ta. longobardicus*, MCSN BES 1018; *Proterosuchus alexanderi*, NMQR 1484) and early rhynchocephalians [144] for which the character can be addressed. As no variation in this character can be addressed in this analysis, we exclude it from this iteration.
— We introduce this character in analysis to account for the anterior concavity in some drepanosauromorph chevrons (e.g. *V. cenensis* (MCSNB 4751), *Me. preonensis* (MFSN 18443)).

139) Epiphyses of limb elements secondary ossification centers: (0) absent, (1) present.

140) Cleithrum: (0) present, (1) absent.

141) Clavicle, ventral articular portion: (0) broader anteroposteriorly than distal portion of clavicle, (1) similar in anteroposterior narrowness to the distal portion of the clavicle.

142) Interclavicle, anterior portion of bone: (0) transversely robust, forming broad diamond; (1) transversely gracile, forming slender, anchor like shape anteriorly.

143) Interclavicle, anterior surface between clavicular articulations: (0) smooth margin, (1) prominent notch in margin.

144) Interclavicle, posterior stem, shape: (0) slender, tapering; (1) marked transverse expansion.

**145) Scapula, scapular blade, shape: (0) flattened blade directed dorsally; (1) flattened blade with large posterior concavity; (2) anterodorsally curved blade.**

— In Pritchard *et al.* [68] and Nesbitt *et al.* [10], this character includes the first two states listed here. We introduce a third state here to describe scapular blades that curve anterodorsally, as in drepanosauromorphs (e.g. *Me. preonensis*, MFSN 1769; *V. cenensis*, MCSNB 4751).

146) Scapula, margin dorsal to glenoid fossa: (0) bears prominent tubercle; (1) smooth bone, lacking tubercle.

147) Coracoid ossifications, number: (0) two, (1) one.

148) Coracoid, infraglenoid morphology: (0) no development of coracoid posteroventral to glenoid, (1) prominent post glenoid process on coracoid, terminating in thickened margin.

149) Sternum, ossification of sternal plates: (0) absent, (1) present.

150) Humerus, ectepicondyle, radial nerve groove: (0) absent, (1) present.

151) Humerus, ectepicondyle, radial nerve groove: (0) no roof, (1) roof present, forming ectepicondylar foramen.

152) Humerus, ectepicondyle: (0) present as prominent preaxial crest, (1) absent, no crest.

153) Humerus, entepicondylar foramen: (0) entepicondylar foramen absent, (1) entepicondylar foramen present.

154) Humerus, entepicondyle, morphology: (0) smooth margin between shaft and postaxial condyle, (1) prominent entepicondylar crest present.

155) Humerus, entepicondylar crest, proximal margin, morphology: (0) crest exhibits a curved proximal margin, (1) crest exhibits a prominently angled proximal margin.

156) Humerus, distal condyles, morphology: (0) distinct trochlear and capitular articulations, (1) low double condyle.

157) Ulna, ossified olecranon process: (0) present, (1) absent.

158) Medial centrale of manus: (0) absent, (1) present.

159) Distal carpal five: (0) absent, (1) present.

160) Manual intermedium: (0) present, (1) absent.

161) Ulnare and intermedium, perforating foramen between elements: (0) present, (1) absent.

162) Manual digit four, phalangeal formula: (0) five phalanges, (1) four phalanges.

163) Pelvis, puboischiadic plate, fenestration: (0) no fenestra, (1) thyroid fenestra within plate.

**164) Ilium, iliac blade, long axis, orientation: (0) horizontal orientation, (1) posterodorsal orientation, (2) anterodorsal orientation.**

— The third state here is added to describe the distinctive shape of the iliac blade of Drepanosauromorpha.

165) Ilium, anteroventral process extending from anterior margin of pubic peduncle: (0) absent, (1) present, process draping across anterior surface of pubis.

166) Ilium, supra-acetabular crest: (0) absent, posterodorsal margin of acetabulum similar in development of anterodorsal margin, (1) prominent anterodorsal bony lamina frames the anterodorsal margin of the acetabulum.

167) Ilium, supra-acetabular surface: (0) dorsalmost margin of acetabulum is unsculptured, (1) prominent bulbous rugosity superior to acetabulum.

168) Ilium, acetabulum, lateral surface: (0) irregular, marked by posterodorsal invasion by finished bone; (1) roughly circular, no posterodorsal invasion.

169) Ilium, iliac blade, anterior surface: (0) smooth anterior margin, (1) anteriorly projecting process or tuber present.

170) Ilium, anterior process/tuber: (0) small, with anterodorsal margin of ilium curving smoothly into dorsal margin of iliac blade; (1) large and anteriorly projecting, with dorsal margin of tuber nearly continuous with dorsal margin of iliac blade.

171) Ilium, posterior process, anteroposterior length: (0) weakly developed failing to extend well posterior of acetabulum, (1) strongly developed extending well posterior to the acetabulum.

172) Ilium, iliac blade, dorsal margin: (0) smoothly textured dorsal border, (1) marked by distinct dorsoventral striations running from acetabulum to dorsal margin of iliac blade.

173) Pubis, symphysis: (0) pubic apron present with distinct anteroventral downturn of the symphyseal region; (1) pubic apron absent, symphysis sits only in coronal plane.

174) Pubis, pubic tubercle: (0) absent, with anterolateral surface of pubis unexpanded; (1) present, with anterolateral surface of pubis expanded into anteroposteriorly broadened tuber.

**175) Pubis, anterolateral surface: (0) lateral pubic tubercle (*sensu* [**55**]) present, manifesting as rounded tuberosity; (1) prominent, transversely narrow ambiens flange present; (2) anterolateral surface of pubis marked by rugose bone.**

— In the initial formulation of this character, Pritchard *et al.* [68] and Nesbitt *et al.* [10] only describe the presence and absence of the lateral pubic tubercle as described by [55]. Further study suggests that this rounded structure is likely homologous to the flattened ambiens muscle attachment in many early Sauria (e.g. *Pr. broomi*, BP/1 2676; *Az. madagaskarensis*, Nesbitt *et al.* 2015). As such, we have added additional states to describe the diversity of conditions in Diapsida.

176) Ischium, posterior margin: (0) vertical and flattened, (1) posterior process extends from posterodorsal margin of bone (spina ischii *sensu* [159]).

177) Femur, profile in preaxial view: (0) sigmoidal curvature, (1) linear shaft with slight ventrodistal curvature.

178) Femur, proximal surface: (0) well ossified, convex; (1) concave surface with central groove.

179) Femur, internal trochanter, proximal portion: (0) crest does not reach femoral head; (1) crest reaches far proximally, continuous with proximal articular surface.

180) Femur, distal condyles, relative size: (0) medial and lateral condyles subequal in transverse/proximodistal dimensions; (1) condyles unequal, lateral condyle larger than the medial condyle.

181) Femur, distal condyles, dimensions relative to femoral shaft: (0) distinct expansion beyond the circumference of the femoral shaft. (1) limited expansion beyond the circumference of the femoral shaft.

182) Femur, tibial condyle: (0) medial surface is rounded and mound like, (1) medial surface is triangular and sharply pointed.

183) Femur, fibular condyle, ventral surface: (0) flattened and planar, (1) rounded and mound like.

**184) Pedal centrale: (0) absent as distinct ossification, (1) present as distinct ossification.**

— In Pritchard *et al.* [68] and Nesbitt *et al.* [10], this character included fusion to the astragalus as a part of state 0. We have removed this statement as it adds an assumption that can only be supported through ontogenetic series or histological work on diapsid astragali.

185) Proximal tarsals (astragalus and calcaneum), co-ossification: (0) present as distinct ossifications, (1) co-ossified.

186) Proximal tarsals (astragalus and calcaneum), perforating foramen: (0) present, situated between astragalus and calcaneum, (1) absent.

187) Calcaneum, distal facet: (0) little broader in dorsal-plantar dimensions than proximal facet; (1) distal facet is markedly expanded in dorsal-plantar dimensions, more than twice the breadth of the proximal facet.

188) Calcaneum, lateral margin: (0) terminates in unthickened margin, (1) roughened tuberosity present laterally.

189) Calcaneum, lateral margin, transverse dimension: (0) little postaxial expansion; (1) markedly broadened, lateral wing of calcaneum twice as broad or broader than the distal calcaneal facet.

190) Calcaneum, lateral projection, ventrolateral margin: (0) coplanar with dorsolateral margin of projection, (1) ventrolateral margin of calcaneum curls externally.

191) Distal tarsal four, proximal surface: (0) smooth contact surface for proximal tarsals, (1) prominent process for contact with proximal tarsals.

192) Pedal centrale, contact with tibia: (0) absent, (1) present.

193) First distal tarsal: (0) present, (1) absent.

194) Second distal tarsal: (0) present, (1) absent.

195) Fifth distal tarsal: (0) present, (1) absent.

196) Metatarsal five, proximal, postaxial: (0) smooth, curved margin; (1) prominent pointed process (outer process *sensu* Robinson 1975) present.

197) Metatarsal five, distal shaft, angle relative to proximal tarsal articulation: (0) straight, with proximal tarsal articulation forming straight line with primary shaft; (1) ‘hooked’, with proximal tarsal articulation forming right angle with primary shaft.

198) Metatarsal five, concavity along preaxial margin; (0) present, (1) absent, metatarsal five blocky in shape.

199) Pedal digit five, proximal phalanx: (0) shorter than proximal phalanx of digit four; (1) proximal phalanx elongate, longer than all other proximal phalanges.

200) Heterotopic ossifications: (0) absent in a minimum of five individuals, (1) present.

201) Maxilla, medial surface dorsal to tooth row: (0) smooth, (1) prominent anteroposteriorly oriented ridge present.

202) Maxilla, dorsal process, shape: (0) posteriorly concave margin, (1) simply tapers to point dorsally.

203) Maxilla, anterolateral surface: (0) large anteriorly opening foramen present, positioned just anterodorsal to primary row of neurovascular foramina; (1) foramen absent.

204) Maxilla, anteromedial surface, palatal process: (0) absent; (1) present, but fails to reach the midline; (2) present and touches its antimere at the midline.

205) Jugal, anterior process: (0) slender and tapering, (1) broad and expanded anteriorly.

206) Ectopterygoid, articulation with the pterygoid: (0) contacts part but not entirety of lateral edge of pterygoid, (1) contacts entire lateral edge of pterygoid.

207) Quadrate, proximal portion, posterior side: (0) continuous with the shaft, (1) expanded and hooked.

208) Parabasisphenoid, orientation of long axis: (0) horizontal, (1) more vertical.

209) Parabasisphenoid, semilunar depression on the lateral surface of the basal tubera: (0) present, (1) absent.

**210) Dentary, posteroventral portion: (0) just meets the angular, (1) laterally overlaps the anteroventral portion of the angular (posteroventral process *sensu* Ezcurra [**63**]).**

— Added terminology from [63].

211) Dentition, crown height of the upper dentition compared with lower dentition: (0) similar tooth crown height, (1) upper dentition is shorter relative to taller lower dentition.

212) Antorbital fossa: (0) restricted to the lacrimal; (1) restricted to the lacrimal and dorsal process of the maxilla; (2) present on the lacrimal dorsal process of the maxilla and the dorsal margin of the posterior process of the maxilla, the ventral border of the antorbital fenestra.

213) Anterior cervical vertebrae (presacral vertebrae 3–5): (0) postzygapophyses separated posteriorly, (1) connected through a horizontal lamina (=transpostzygapophyseal lamina) with a notch at the midline.

***214) Cervical vertebrae ratio of lengths of fourth or fifth cervical centra to heights of anterior articular surfaces: (0) less than 1, (1) 1–3, (2) 3–10, (3) greater than 10. ORDERED.***

— Character modified from Nesbitt *et al.* [10], which only described states for cervical centra with lengths greater than heights and heights greater than lengths. Ezcurra [63] described additional states for the relative lengths of anterior cervical centra. I have provided these states with consideration for the ratios apparent in the sample of taxa present in this specific analysis.

215) Dorsal vertebrae, diapophysis, anteroposteior position: (0) anterior portion of the neural arch and/or centrum, (1) anteroposterior middle of the neural arch and/or centrum.

216) Sacral ribs, anteroposterior length of first primordial sacral rib versus second primordial sacral rib: (0) longer anteroposteriorly than primordial sacral rib two, (1) about the same length or longer anteroposteriorly than second primordial sacral rib.

217) Anterior caudal vertebrae, neural spines: (0) inclined posteriorly, (1) vertical.

218) Caudal vertebrae, length of the anterior caudal vertebrae (caudal vertebrae 1–10) relative to posterior caudal vertebrae (approx. 25): (0) nearly the same length, (1) posterior caudal vertebrae much longer.

219) Scapula, anterior margin: (0) straight or partially concave, (1) markedly concave.

***220) Scapula, scapular blade, ratio of dorsoventral height to anteroposterior length at base of blade: (0) less than 0.4, (1) 0.4–0.25, (2) greater than 0.25. ORDERED.***

— This character in Nesbitt *et al.* [10] included states only describing this ratio being greater or less than 0.25. We have integrated new states to describe the extreme elongation in drepanosauromorphs more derived than *Hy. limnaios*, *Teraterpeton* and *Trilophosaurus*, which were coded as having a ratio of less than 0.25 have been remeasured and are found to fall in state 1 for this analysis.

221) Humerus, distal end, transverse width: (0) less than 2.5 times the minimum width of the shaft, (1) equal to or more than 2.5 times the minimum width of the shaft.

222) Manual ungual, length: (0) about the same length or shorter than the penultimate phalanx of the same digit, (1) distinctly longer than penultimate phalanx of the same digit.

223) Ilium, acetabulum, ventral margin: (0) convex, (1) concave.

224) Ilium, iliac blade, maximum anteroposterior length: (0) less than three times maximum dorsoventral height, (1) more than three times maximum dorsoventral height.

225) Ischium, anteroposterior length: (0) about same length or shorter than dorsal margin of iliac blade, (1) markedly longer than dorsal margin of iliac blade.

226) Femur, ridge of attachment of the *M. caudifemoralis*: (0) bladelike with a distinct asymmetric apex located medially (=internal trochanter), (1) low and without a distinct medial asymmetrical apex (=fourth trochanter).

227) Femur, anterior trochanter (*M. iliofemoralis cranialis* insertion): (0) absent, (1) present.

228) Astragalus, tibial and fibular articulations: (0) separated by a gap or notch of Gower 1996, (1) continuous.

229) Calcaneum calcaneal tuber (=primordial lateral projection in early diapsid groups), shaft proportions at the midshaft of the tuber: (0) taller than broad, (1) about the same or broader than tall.

230) Calcaneum, articular surfaces for fibula and distal tarsal IV: (0) separated by a nonarticular surface, (1) continuous.

231) Calcaneum, tuber (=primordial lateral projection in early diapsid groups), orientation relative to the transverse plane: (0) lateral, less than 20°; (1) posteriorly deflected, between 21° and 49°; (2) posterolaterally between 50° and 90° posteriorly. ORDERED.

232) Metatarsal IV, proximodistal length: (0) longer than metatarsal III, (1) about the same length as or shorter than metatarsal III.

233) Pes, unguals, ventral tubercle: (0) absent or small, (1) well developed and extended ventral to proximal articular facet of ungual.

234) Distal non-ungual pedal phalanges, distal articular portion: (0) lateral and medial sides parallel or near parallel, (1) lateral and medial sides converging anterodorsally.

235) Pes, penultimate phalanx, proximodistal length: (0) shorter than the more proximal phalanx, (1) significantly longer than the more proximal phalanx.

236) Osteoderms: (0) absent, (1) present.

237) Prefrontal, orbital margin: (0) lateral surface smooth or with slight grooves; (1) rugose lateral sculpturing present.

238) Gastralia: (0) abundant, with individual gastral elements nearly contacting medially; (1) small in number, well separated, or unossified.

— This character was included in Nesbitt *et al.* [10] along with Character 137 the original gastralia presence/absence character of Pritchard *et al.* [68]. The Pritchard *et al.* [68] character is removed in this study to avoid overprinting.

239) Astragalus, margin between tibial and fibular facets: (0) grades smoothly into anterior hollow of astragalus, (1) prominent ridge separates margin from anterior hollow.

***240) Proximal tarsals, morphology of perforating foramen: (0) broad, marked by finished bone on astragalus and calcaneum; (1) pinched, marked by extremely constricted space between astragalus and calcaneum.***

— The original Character 140 in Nesbitt *et al.* [10] described the anterior process of the chevrons in some early archosauromorphs (e.g. *Tr. buettneri*). However, this morphology was also incorporated into Character 130 of Pritchard *et al.* [68], resulting in overprinting of this morphology. We remove the character in Nesbitt *et al.* [10] and retain the character state within Character 138.
— This character describes the ‘pinched’ morphology of the perforating foramen in a number of early diapsids (e.g. *Pe. kansensis* [131]); *Ta. longobardicus*, MCSN V 3730; *Tanytrachelos ahynis*, Pritchard *et al.* [68]) in contrast to the well-defined opening between the tarsals in most other taxa (e.g. *Pr. broomi*, BP/1 2676; *Proterosuchus* sp., AMNH FARB 2237).

241) Dentary, anterior portion: (0) in same horizontal plane as anteroposterior middle portion of dentary, (1) anteroventrally deflected relative to anteroposterior middle portion of dentary.

242) Quadrate, posterior margin, ventral half: (0) flat or slightly concave, (1) distinctly convex.

243) Atlas, centrum: (0) separate from axial intercentrum, (1) fused to axial intercentrum.

244) Axis, neural spine: (0) dorsal margin inclined anteroventrally, (1) dorsal margin inclined anterodorsally.

245) Presacral vertebrae (fifth vertebra to the sacrum), neural arch, posterior edge: (0) spinopostzygapophyseal laminae absent, (1) spinopostzygapophyseal laminae present.

**246) Dentary, lateral exposure, posterior extent: (0) posteriormost extent of dentary on dorsum of mandible-posterodorsal process of dentary *sensu* [**63**]; (1) posteriormost extent of dentary positioned ventral to surangular (posterocentral process of dentary *sensu* [**63**]). ORDERED.**

— Terminology added from Ezcurra [63] for the various processes of the dentary.

247) Premaxilla, medial surface, contribution to palate: (0) absent, (1) flattened palatal process present.

***248) Premaxilla, palatal process: (0) extends posteriorly as far as the lateral surface of the premaxilla, (1) extends posteriorly past the lateral surface of the premaxilla.***

— The remaining characters in this analysis are novel additions to the Pritchard *et al.* [68] and Nesbitt *et al.* [10] datasets. Some of these characters and codings are derived from Pritchard *et al.* [11].
— This character describes the relative anteroposterior length of the palatal process of the premaxilla, which is extensive in some early archosauromorphs. In South African *Proterosuchus* (e.g. NMQR 880, 1484) and ‘*Ch.*’ *yuani* (cast of IVPP V 36315), the palatal process of the premaxilla extends posteriorly past the premaxillary tooth row, paralleling the lateral margin of the maxilla. The palatal process in most archosauriforms is subequal in length to the dentigerous portion of the premaxilla (e.g. *B. kupferzellensis* [98], *E. africanus* (NHMUK R3592)).

249) Premaxilla, fusion to contralateral premaxilla: (0) absent, (1) present.

— Derived from similarly informative Character 62 in [70].

250) Premaxilla, tooth morphology: (0) similar in morphology to maxillary teeth, (1) single, apicobasally elongate, chisel-like tooth, (2) teeth longer than maxillary teeth with subcircular cross section.

— State 1 describes the unique shape of the premaxillary teeth in some derived Sphenodontia. Similar states occur in Evans [73], Character K11; Gauthier *et al.* [70], Character 55; Wu [160], Character 25; and Rauhut *et al.* [161], Character 52. State 2 describes the apicobasally elongated teeth in some early archosauromorphs (e.g. *Az. madagaskarensis*, [67]; ‘*Ch.*’ *yuani*, cast of IVPP V 36315).

251) Maxilla, dentigerous surface, ventral surface: (0) ungrooved; (1) single, anteroposteriorly running groove; (2) two anteroposteriorly running grooves. ORDERED.

— Derived from similarly informative characters in Dilkes [122], Character 9; and Dilkes [33], Character 64.

252) Nasals, anterior margins: (0) appressed nasals form anteriorly pointed structure in midline, (1) appressed nasals form anteriorly flattened surface, (2) appressed nasals are separated anteriorly by premaxilla.

— Derived from similarly informative characters in Dilkes [33], Character 13; Ezcurra *et al.* [89] Character 127; and Ezcurra [63], Character 78.

253) Lacrimal: (0) present as distinct ossification, (1) absent.

— Derived from similarly informative characters in Evans [73], Character K3; Gauthier *et al.* [70], part of Character 1; and Rauhut *et al.* [161], Character 9. Note that taxa coded as ‘1’ for this character are coded as ‘-’ for Characters 11 and 12.

254) Postfrontal, shape of dorsal exposure: (0) forms a right triangle with right angle forming posteromedial margin, (1) anteroposteriorly broad, posterior margin inclined posteromedially.

— This character describes the shape of the dorsal exposure of the postfrontal bone in Diapsida. In a number of taxa (e.g. *Petrolacosaurus* (Reisz, 1981), *Pr. broomi* (UCMP 37151) and *Proterosuchus fergusi* (SAM PK 10603)), the posterior margin of the postfrontal is medially directed. However, the posterior margin of the bone is posteromedially inclined in some early diapsids (e.g. *Y. capensis* (BP/1 3393), *Ho. boulei* [121]), archosauromorphs (e.g. *Az. madagaskarensis* (UA 7-20-99-653), *Tr. buettneri* (TMM 31025-140)) and lepidosaurs (e.g. *Cle. hudsoni* [110], *Sp. punctatum* [145]). This character relates to Character 27, which describes the absence of a postorbital–parietal contact due to the postfrontal. However, there are a number of taxa with anteroposteriorly broad postfrontals that still exhibit a postorbital–parietal contact (e.g. *Tr. buettneri* (TMM 31025-140)).

255) Parietal, posterolateral (=post-temporal) process: (0) slender and tapering; (1) anteroposteriorly flattened, such that parietal contributes prominently to occipital face of skull.

— Derived from Ezcurra [63], Character 168.

256) Parietal, posterolateral (=post-temporal) process: (0) lateral ornamentation absent margin is smooth; (1) ornamentation present, margin marked by dorsolaterally oriented row of pointed projections.

— Novel character.
— In both species of *Rautiania* ([162]; PIN 5130/1, 5130/2) and *Weigeltisaurus jaekeli* ([163]; SMNS 53439), the parietal is marked by a series of laterally oriented spines. This feature is absent in all other known diapsids and the weigeltisaurid *Coelurosauravus elivensis* ([164]; MNHN MAP 325).

257) Skull roof, upper temporal fenestra: (0) absent, dorsal skull roofing bones cover adductor chamber; (1) present as gap between dorsal exposures of skull roofing bones.

— Derived from similarly informative characters in Gauthier *et al.* [70], Character 13; Rieppel [5], Character 10; and Müller [94], Character 9.

258) Skull roof, posterolateral surface, ornamentation: (0) absent, (1) prominent horns on squamosal and quadratojugal.

— Novel character.
— In weigeltisaurid diapsids (e.g. *Co. elivensis*, *W. jaekeli*), the lateral surfaces of the post-temporal skull bones are marked by laterally oriented spines (e.g. [164,165]).

259) Quadrate, pterygoid ramus, ventral margin of posterior base of ramus: (0) in line with quadrate condylar surface, (1) elevated dorsally relative to quadrate condylar surface.

— Novel character.
— In the diapsids *Pe. kansensis* [131], CQ drepanosaurid (AMNH FARB 30834), and *Rautiania* sp. (PIN 5130/33), the ventral margin of the pterygoid ramus extends ventrally to the level of the condylar surface of the quadrate. In *Y. capensis* (AMNH FARB 5561), early archosauromorphs (e.g. *Pr. broomi* (UCMP 37151), *M. bassanii* (MCSN V 457)) and lepidosauromorphs (e.g. *Cle. hudsoni* [18]), the ventral margin of the quadrate ramus is elevated dorsally relative to the quadrate condyles.

260) Suborbital fenestra: (0) absent, no gap between palatine ectopterygoid and pterygoid; (1) present, cavity on the palate present between palatine ectopterygoid and pterygoid.

— Derived from similarly informative characters in Gauthier *et al.* [166], Character 33; and Müller [94], Character 31.

261) Palatine, posterolateral portion, transverse expansion: (0) absent, producing anteriorly curved suborbital fenestra; (1) present, producing anteriorly tapered suborbital fenestrae.

— Derived from similarly informative characters in Wu [160], Character 33; Apesteguia & Novas [167], Character 22; and Rauhut *et al.* [161], Character 23.
— Taxa coded as ‘0’ for Character 260 are coded as ‘-’ for this character.

262) Prootic, foramen for facial nerve, lateral exit: (0) sits within broad open surface along anterior inferior process, (1) exit sandwiched between crista prootica anterodorsally and an additional thin plate of bone posteroventrally.

— Novel character.
— In most early archosauromorphs and non-saurian diapsids, the lateral foramen for the facial nerve is situated posterior to the crista prootica on the flat, lateral surface of the prootic (e.g. *Az. madagaskarensis*, FMNH PR 2765; *Cz. harae* [56]). In a number of archosauriforms, the crista prootica is appressed posteriorly to an additional crest of bone. This has the effect of ‘sandwiching’ the facial nerve foramen between these two bone crests. This condition may be seen in *Proterosuchus fergusi* (BP/1 3393), *E. africanus* (NHMUK R 3592), *Fugusuchus hejiapenensis* [63,168] and *Xilousuchus sapingensis* [168,169].

263) Parabasisphenoid, cultriform process, dentition: (0) teeth run anteroposteriorly on process, (1) teeth clustered at base of process.

— Novel character.
— In those early amniotes that bear teeth on the parasphenoid rostrum, the teeth run anteroposteriorly along the ventral surface of the structure, as well as being clustered at the base of the process. This condition occurs in *Paleothyris acadiana* [170], *Pe. kansensis* [131] and *Lanthanolania ivakhnenkoi* [171].

264) Stapes, stapedial shaft, robusticity: (0) robust with thick shaft, similar or greater in breadth to the paroccipital process of the opisthotic; (1) slender with rod-like shaft, much slenderer than paroccipital process.

— Derived from similarly informative characters in Gauthier *et al.* [70], Character 35; deBraga & Rieppel [172], Character 45; and Müller [94], Character 133.

265) Surangular, dorsolateral surface: (0) transversely narrow, (1) exhibits transversely wide shelf.

— Derived from similarly informative characters in deBraga & Rieppel [172], Character 87; Müller [94], Character 166; Ezcurra *et al.* [173], Character 163; and Ezcurra [63], Character 286.

266) Articular, fossa posterior to glenoid: (0) subequal or greater in anteroposterior length to the glenoid fossa; (1) anteroposteriorly constricted, shorter than the glenoid fossa.

— The retroarticular process in most early Sauria exhibits a dorsally concave basin that likely contributed support for the tympanic membrane. In most early saurians, this basin is similar in anteroposterior elongation to the glenoid fossa itself (e.g. *Tr. buettneri* (TMM 31025-140), *M. bassanii* (PIMUZ T.2472), *Mes. browni* (SAM PK 6536)). In early archosauriforms, the basin posterior to the glenoid is anteroposteriorly shorter than that fossa. This may be seen in *Proterosuchus fergusi* (BP/1 3393), *E. africanus* (NHMUK 3592) and *B. kupferzellensis* (SMNS 80260).

267) Articular, retroarticular process, dorsoventral dimensions: (0) shallow, dorsal margin positioned posteroventral to quadrate articulation; (1) deep, dorsal margin at dorsoventral level equivalent to quadrate articulation.

— In many early diapsids and lepidosauromorphs, the retroarticular process is dorsoventrally short, with its dorsal margin sloping ventrally relative to the quadrate articulation of the articular. This condition occurs in *Y. capensis* (AMNH FARB 5561), *Me. preonensis* (MFSN 1769), *Cla. germaini* [106] and *Sp. punctatum* [145]. By contrast, the retroarticular process of the CQ drepanosaurid and most archosauromorphs is dorsoventrally higher, with its dorsal margin at a level equivalent to the quadrate articulation. In archosauromorphs, this condition occurs in *Protorosaurus speneri* (USNM 442453, Gottmann-Quesada and Sander, 2009), *M. bassanii* (PIMUZ T.2477), *Tr. buettneri* (TMM 31025-140) and ‘*Ch.*’ *yuani* (IVPP V4067).

268) Articular, retroarticular process, posterior tip: (0) oriented posteriorly oriented, (1) posterodorsally upturned.

— Derived from similarly informative characters in Dilkes [33], Character 75; Ezcurra *et al.* [173], Character 167; and Ezcurra [63], Character 284.

269) Axis intercentrum, fusion to axial centrum: (0) absent, (1) present.

— In nearly all early diapsids and saurians, the axial intercentrum and centrum are unfused to one another throughout ontogeny. This occurs in *Pe. kansensis* [131], *Y. capensis* (AMNH FARB 5561), *Ta. longobardicus* (PIMUZ T.2819) and *Az. madagaskarensis* [10]. However, in some lepidosaurs and archosauromorphs, these bones are fused. Examples occur in *G. bridensis* [119], *Pl. robinsonae* [174] and *Sp. punctatum* (YPM R 10646 [175]).

270) Postaxial cervical vertebrae, intervertebral articulations: (0) circular or ovoid articular surfaces appressed to one another directly, (1) saddle-shaped articular surface (=heterocoely).

— This character describes the saddle-shaped intervertebral articulations of the cervical centra in drepanosauromorphs (e.g. *Me. preonensis*, MPUM 6008; vertebral material from Cromhall Quarry [27]; CQ drepanosaurid, AMNH FARB 30834).

271) Anterior cervical vertebra, postzygapophysis, epipophysis, morphology: (0) form vertical expansions of bone above facet but do not extend posteriorly beyond facet, (1) form posteriorly pointed projections that barely project posteriorly beyond the level of the facet, (2) form posteriorly pointed projections that project far posteriorly beyond the level of the facets. ORDERED.

— A number of prior analyses have described the presence of prominent bony expansions on the dorsal surfaces of the postzygapophyses of cervical vertebrae (e.g. [68,136]).

272) Anterior cervical vertebra, hypapophysis: (0) absent, ventral surface of centrum unexpanded, (1) posteroventrally posteroventral surface of centrum exhibits massive posteroventrally projecting crest.

— Novel character.
— In most early diapsids and saurians, the ventral surface of anterior cervical centra is marked only by a midline keel (e.g. *Pr. broomi*, BP/1 2675; *Tr. buettneri*, TMM 31025-140). The posteroventral margin of the centrum possesses a posteroventrally oriented bony projection in drepanosaurids (e.g. *Me. preonensis*, MFSN 1769; CQ drepanosaurid, AMNH FARB 30834) and some squamates (e.g. *Sh. crocodilurus* [138]).

273) Cervical ribs: (0) present as distinct ossifications from cervical vertebrae, (1) absent as distinct ossifications.

— Derived from Renesto *et al.* [27], Character 5.

274) Anterior dorsal vertebra, neural arch, surface ventrolateral to base of neural spine: (0) smooth, (1) surface marked by deep concavity.

— Derived from similarly informative characters in Dilkes [33], Character 84; Müller [94], Character 103; and Ezcurra *et al.* [173], Character 184.

275) Anterior dorsal vertebra, pedicel, dorsoventral height: (0) substantially shorter than respective centra, (1) taller than respective centra.

— Derived from Pritchard *et al.* [11], Character 52.

276) Anterior dorsal vertebra, neural spine, anteroposterior expansion: (0) remain roughly similar in anteroposterior length throughout dorsoventral height; (1) dorsally broader anteroposteriorly than at spine base; (2) third dorsal spine anteroposteriorly expanded into hatchet shape, contacting other dorsal neural spines. ORDERED.

— Derived from Renesto *et al.* [27], Characters 7 and 9.

277) Dorsal ribs, accessory ossification at distal tip: (0) absent, (1) present.

— Novel character.
— In Weigeltisauridae, the ribs of the dorsal region contact an additional, proximodistally elongate ossification that likely formed the framework for the patagium. This is seen in *W. jaekeli* [165] and *Co. elivensis* (MNHN MAP 327). These structures have not been reported from the isolated remains of *Rautiania* (e.g. [162,176]).

278) Mid-dorsal ribs, fusion to respective centra: (0) absent, (1) present.

— Derived from Renesto *et al.* [27], Character 11.

279) Dorsal ribs, curvature of rib shaft: (0) curve ventromedially to frame trunk; (1) splayed laterally, forming patagium.

— Novel character.
— In Kuehneosauridae, the ribs of the dorsal region are straight, splaying out from the midline. This construction is hypothesized to form the patagium (e.g. [123,124]) and may be seen in *Kuehneosaurus* spp. [124] and *I. siefkeri* (AMNH FARB 2101).

280) Anterior caudal vertebra, neural spine, dorsoventral height: (0) similar in height or shorter than sacral neural spines, (1) taller than sacral neural spines.

— Derived from similarly informative characters in Dilkes [33], Character 88; and Ezcurra *et al.* [173], Character 189.

281) Caudal vertebra, anterior neural spine, anteroposterior expansion of spine tip: (0) unexpanded dorsally, (1) slender anterior and posterior projections forming T-shape.

— Derived from similarly informative characters in Senter [36], Character 36; Renesto *et al.* [27], Character 33.

282) Chevron, proximal articular morphology: (0) unfused to centra, (1) fused to centra.

— Derived from Pritchard *et al.* [11], Character 49.

283) Anterior chevron, hemal spine, morphology: (0) forms single spine, (1) bifurcates ventrally.

— Derived from similarly informative characters in Senter [36], Character 41; and Renesto *et al.* [27], Character 37.

284) Anteriormost chevron, hemal spine, morphology: (0) bifid spines remain separate ventrally, (1) bifid spines recontact ventrally forming foramen.

— Derived from similarly informative characters in Senter [36], Character 41; and Renesto *et al.* [27], Character 37.
— Taxa coded as ‘0’ for Character 283 are coded as ‘-’ for this character.

285) Posterior chevron, proximal articulation: (0) positioned intervertebrally, (1) positioned at anteroventral surface of centrum.

— Derived from similarly informative characters in Renesto *et al.* [27], Character 39.

286) Terminal caudal vertebra(e): (0) similar in morphology to other posterior caudals, (1) modified into claw-like element.

— Derived from similarly informative characters in Senter [36], Character 37; and Renesto *et al.* [27], Character 40.

287) Supraneural ossification (bone growth positioned anterodorsal to anterior dorsal neural spines): (0) absent, (1) present.

— Derived from Pritchard *et al.* [11], Character 73.

288) Scapulocoracoid, glenoid fossa, position: (0) at or near base of scapular blade, (1) located far ventral of base of scapular blade.

— Derived from Pritchard *et al.* [11], Character 71.

289) Scapulocoracoid, glenoid fossa, orientation relative to long axis of trunk: (0) oriented posterolaterally, ventral margin extends posterior of dorsal margin; (1) oriented laterally, ventral margin positioned directly underneath to dorsal margin.

— Derived from Pritchard *et al.* [11], Character 74.

290) Humerus, internal tuberosity: (0) continuous with humeral shaft, (1) offset from humeral shaft by cylindrical pedicel of finished bone.

— Ezcurra [63] E Character 421 describes the presence or absence of ‘[h]umerus, internal tuberosity distinctly separated from the proximal articular surface in anterior or posterior views’. Our character differs from this definition, in that the tuberosity must not only be displaced from the proximal humerus but also placed on a pedicel. This condition may be seen in *K. latus* (AMNH FARB 7784) and *I. siefkeri* (AMNH FARB 2101).

291) Humerus, epicondyles, proximal base: (0) positioned distal to midshaft, (1) positioned at near midshaft.

— Derived from Pritchard *et al.* [11], Character 72.

292) Humerus, ectepicondyle, morphology of lateral margin: (0) squared off preaxially, (1) pointed triangular preaxially.

— Novel character.
— In most early diapsids and archosauromorphs with a well-defined ectepicondyle, the lateral margin of the structure is linear. This can be seen in *D. unguicaudatus* (MCSNB 5728), *Sh. crocodilurus* [138] and *Sp. punctatum* [144]. In *K. latus* (AMNH FARB 7786) and *I. siefkeri* (AMNH FARB 2101), the ectepicondyle is triangular, with a laterally oriented apex.
— Taxa coded as ‘1’ for Character 152 are coded as ‘-’ for this character.

293) Humerus, entepicondyle, distal extent: (0) terminates proximal to the distal margin of the ulnar condyle, (1) extends distally relative to ulnar condyle.

— In most diapsid reptiles, the distal margin of the entepicondyle of the humerus is roughly equivalent in distal extent to the distal margin of the ulnar condyle. In weigeltisaurids, the distal end of the entepicondyle is expressed further distally than the ulnar condyle. This condition may be seen in *Co. elivensis* (MNHN MAP 317) and *Rautiania* sp. [176].
— Taxa coded as ‘0’ for Character 154 are coded as ‘-’ for this character.

294) Humerus, distalmost end: (0) collinear with proximal shaft, (1) primary axis of shaft curves towards flexor surface at distal end.

— Derived from Pritchard *et al.* [11], Character 69.

295) Radius, proximal tab: (0) absent, (1) prominent tab for articulation with ulna present.

— Derived from Pritchard *et al.* [11], Character 56.

296) Radius, distal articulation: (0) terminal surface is concave, (1) small styloid process on radius fits into radiale.

— Derived from similarly informative characters in Gauthier *et al.* [70] Character 99, which describes the unique articulation between radius and radiale in squamates. A very similar character state is evident in Chinle Formation *Drepanosaurus* sp. [11].

297) Ulna, shape: (0) similar to radius with elongate shaft, (1) flattened in preaxial/postaxial plane forming enormous crescent.

— Derived from Renesto *et al.* [27], Character 18; and Pritchard *et al.* [11], Character 55.

298) Ulnare and intermedium, proximodistal elongation: (0) longer proximodistally than in preaxial–postaxial plane; (1) elements short, equivalent in proximodistal and preaxial–postaxial length.

— Derived from Pritchard *et al.* [11], Character 61.

299) Second manual ungual: (0) similar in morphology to other manual, (1) substantially taller and more massive than other manual unguals.

— Derived from Pritchard *et al.* [11], Character 71.

300) Manual digit three III, phalangeal formula: (0) multiple phalanges, (1) single non-ungual phalanx.

— Derived from Pritchard *et al.* [11], Character 62.

301) Ilium, anteroventral margin of iliac blade: (0) inclined posterodorsally, (1) inclined vertically, (2) inclined anterodorsally. ORDERED.

— Novel character.
— In a most early diapsids and Lepidosauria, the anterior margin of the iliac blade anterodorsal to the acetabulum is oriented posterodorsally. This condition occurs in *Pe. kansensis* [131], *Th. colcanapi* (MNHN MAP 360) and *Sh. crocodilurus* [138]. In some early archosauromorphs, the anterior margin of the iliac blade is vertically oriented (e.g. *Tr. buettneri* (TMM 31025-73)). In most early archosauromorphs, the anteroventral base of the iliac blade curves anterodorsally, providing a ventral base for an anterior expansion of the blade (e.g. *Ta. longobardicus* (PIMUZ T.1277), *M. bassanii* (MCSN V 457) and ‘*Ch.*’ *yuani* (IVPP V4067)).
— Note that this character is independent of the presence of an anterior tuberosity on the anterior surface of the ilium. Such a tuber is present on the anterior margin of the bone in *Sh. crocodilurus* [138] and *Tr. buettneri* (TMM 31025-73), although this structure does not alter the orientation of the anterior base of the iliac blade.

302) Ilium, iliac blade, post-acetabular portion: (0) relatively planar or lightly sculptured; (1) marked by posterodorsally running ridge, extending from posterior margin of supra-acetabular margin.

— Derived from Ezcurra [63] Character 465, although we only incorporate a single state to describe this structure. We also refrain from describing this ridge as an attachment for *M. caudifemoralis brevis* pending further study of this ridge in early archosauromorphs that possess it (e.g. *Ta. longobardicus* (T.1277), *Pr. broomi* (BP/1 2676)).

303) Femur, proximal surface, dorsal surface: (0) unornamented, (1) marked by prominent tuberosity.

— Novel character.
— In a number of early amniote taxa, the dorsal surface of the proximalmost end of the femur is marked by a large tuberosity. Examples of this condition include *Ca. aguti* (Holmes, 2003), *Ar. gracilis* (MCZ 2043) and *Hy. limnaios* (AMNH FARB 7759). In most other early diapsids and Sauria, the dorsal surface of the femur is unornamented (e.g. *Y. capensis* (BP/1 3859), *Pr. broomi* (BP/1 2676)). For the time being, we do not consider this tuberosity to be homologous with the anterior trochanter of dinosauromorphs, although its homology remains to be determined.

304) Calcaneum, primordial lateral projection, ventral margin: (0) convex and continuous with the lateral margin of the projection; (1) margin is concave, sharply angled relative to lateral margin of the projection.

— Derived from Pritchard *et al.* [11], Character 59.

305) Metatarsal I, shaft, proximodistal length relative to proximodistal length of metatarsal IV: (0) greater than 0.42, (1) 0.42–0.32, (2) less than 0.32. ORDERED.

— Derived from Ezcurra *et al.* [173, p. 216] and Ezcurra [63], Character 569.

306) Pes digit three III, phalangeal formula: (0) four, (1) three.

— Derived from similarly informative characters in Renesto *et al.* [27], Character 28; and Pritchard *et al.* [11], Character 62.

307) Anterior cervical vertebra, neural arch, transverse breadth at anteroposterior midpoint relative to centrum: (0) subequal, (1) substantially broader.

— Novel character.
— In most early diapsids and Sauria, the transverse width of the neural arches of anterior cervical vertebrae is subequal to that of the cervical centrum of the same vertebra (e.g. *Y. capensis* (BP/1 3859), *Pr. broomi* (BP/1 2675), *Proterosuchus alexanderi* (NMQR 1484)). In some drepanosaurids (e.g. *D. unguicaudatus* (MCSNB 5278), CQ drepanosaurid (AMNH FARB 30834)), the neural arches of anterior cervical vertebrae are transversely broader than their respective centra.

**C.4. Methods for phylogenetic analysis, topological constraint analyses and results**

All analyses are run in TNT v. 1.5 [177], employing the ‘Traditional Search’ options including 10 000 replicates of Wagner trees (using random addition sequences), followed by tree bisection and reconnection (TBR) holding 10 trees per replicate. The best trees obtained at the end of the replicates were subjected to a final round of TBR branch swapping. We employed Rule 1 of Coddington & Scharff [178] for collapsing zero-length branches. We designated the Carboniferous diapsid *Pe. kansensis* as the outgroup for this analysis. We employed the STATS.RUN TNT script to obtain the Consistency Index and Retention Index for all trees. We used the Bremer support option in the Trees submenu of TNT, calculating supports based on a new round of TBR, holding trees suboptimal by 15 steps. A nexus file containing the phylogenetic data matrix analysed in this paper is available on Data Dryad [179]. The matrix is also published on Morphobank (www.morphobank.org) as project 2214.

The primary analysis presented in figures 3 and 4 recovered four most-parsimonious trees of 1009 steps in length (CI = 0.336, RI = 0.651) recovered in 4277 out of 10 000 replicates. We also ran a jackknife analysis (10 000replicates, 20% character removal probability), the results of which are presented as GC values (frequency differences). The results of this analysis are presented in figure 8. A stratigraphically calibrated strict consensus is presented in figure 9.

To test the suboptimality of past hypotheses of the affinities of Drepanosauromorpha among Diapsida, we ran several constraint analyses. In each case, we created unresolved trees in which the only resolved clades included (1) all included drepanosauromorphs in an unresolved polytomy and (2) the hypothesized sister clade (or species) in an unresolved polytomy. Our constraint analyses included the following permutations: (i) a sister relationship with Tanystropheidae (hypothesized by [33]), (ii) a sister relationship with *Protorosaurus speneri* (hypothesized for *Me. preonensis* in [34]), (iii) a sister relationship with Weigeltisauridae (hypothesized in an analysis including *Coelurosauravus* and most drepanosauromorph taxa by [36]) and (iv) a sister relationship with Kuehneosauridae (hypothesized by [94]). For each constraint analysis, we used the same search methods described above for the unconstrained analysis.

— Enforcing a sister relationship with Tanystropheidae produced four most-parsimonious trees of 1028 steps in length recovered in 9165 out of 10 000 replicates. This result is 19 steps longer than the most-parsimonious trees in the primary analysis. This analysis recovers a monophyletic Protorosauria in the sense of Dilkes [33]; *Protorosaurus speneri* is the sister taxon of the clade Tanystropheidae + Drepanosauromorpha. Archosauromorpha is substantially less resolved than in the primary analysis. The resulting topology is presented in figure 10.
— Enforcing a sister relationship with *Protorosaurus speneri* produced six most-parsimonious trees of 1024 steps in length recovered in 8738 out of 10 000 replicates. This result is 15 steps longer than the most-parsimonious trees in the primary analysis. In the most-parsimonious trees, *Protorosaurus* + Drepanosauromorpha is the earliest diverging branch within Archosauromorpha following the Archosauria + Lepidosauria divergence. The phylogeny of Archosauromorpha has altered, with *Boreopricea* + Kuehneosauridae being the next divergence, followed by Allokotosauria. The phylogeny of non-saurian diapsids is largely congruent with the primary analysis, although *Claudiosaurus* is now the earliest diverging diapsid rather than *Orovenator*. The resulting topology is presented in figure 11.
— Enforcing a sister relationship with Weigeltisauridae produced 32 most-parsimonious trees of 1011 steps in length recovered in 46 238 of 10 000 replicates. This result is two steps longer than the most-parsimonious trees in the primary analysis. In the resultant topologies, non-saurian diapsids are poorly resolved. The topology of Sauria is identical to the primary analysis. The resulting topology is presented in figure 12.
— Enforcing a sister relationship with kuehneosaurids produced two most-parsimonious trees of 1019 steps in length recovered in 9547 out of 10 000 replicates. This result is 10 steps longer than the most-parsimonious tree in the primary analysis. In the most-parsimonious trees, Drepanosauromorpha and Kuehneosauridae are the sister taxon of *Protorosaurus* + all other Archosauromorpha. The phylogeny remains otherwise similar in its topology, although *Claudiosaurus* is now the earliest-diverging diapsid after *Pe. kansensis*. *Boreopricea funerea* is the sister taxon of *Prolacerta* + Archosauriformes. The resulting topology is presented in figure 13.
