# Supplementary material for: A bird-like skull in a Triassic diapsid reptile increases heterogeneity of the morphological and phylogenetic radiation of Diapsida
Source: R Soc Open Sci. 2017 Oct 11;4(10):170499. doi: 10.1098/rsos.170499 (PMC5666248; doi:10.1098/rsos.170499)
Supplement: Supplemental Appendix A: A. Modifications to characters and codings from the matrix of Nesbitt et al. (2015).; Appendices for Main Text PDF (A–C) [file rsos170499supp1.docx]

**Electronic supplementary materials to**

**“A bird-like skull in a Triassic diapsid reptile increases heterogeneity of the morphological and phylogenetic radiation of Diapsida”**

**Table of Contents**

1. **Modifications to characters and codings from the matrix of Nesbitt et al. (2015).**

**A. Modifications to characters and codings from the matrix of Nesbitt et al. (2015).**

The following are codings changed from the dataset of Nesbitt et al. (2015). Some are corrections from the original dataset, whereas others are revised based on firsthand studies of material that was not incorporated into the Nesbitt et al. (2015) study nor Pritchard et al. (2015). Changes are only included for characters that were unchanged from the original study or changed only slightly (indicated in **bold** above). Characters in **red** were completely newly coded for this study.

1.

*Amotosaurus* ?->0.

2.

*Rhynchosaurus*

*Teyumbaita*

1->2.

3.

*Langobardisaurus*

*Tanystropheus*

*Tanytrachelos*

*Macrocnemus*

0->1.

4.

*Amotosaurus*

*Langobardisaurus*

?->1

5.

*Langobardisaurus*

?->0

8.

*Teraterpeton*

0->1.

*Langobardisaurus*

0->?

*Amotosaurus*

?->0

9.

*Tanystropheus*

?->0

11.

*Uromastyx*

1->?

*Amotosaurus*

*Langobardisaurus*

?->0

15.

*Batrachotomus*

*Euparkeria*

*Erythrosuchus*

*Proterosuchus* South African

*Proterosuchus yuani*

1->2 (new state)

16.

*Gephyrosaurus*

0->1

19.

*Tanystropheus*

0->1.

20.

*Rhynchosaurus*

1->2.

26.

*Uromastyx* (fused postorbitofrontal).

1->”-“

*Teraterpeton*

1->0.

28.

*Gephyrosaurus*

*Shinisaurus*

*Tanytrachelos*

0->?

*Uromastyx*

?->1.

*Azendohsaurus mad*

*Euparkeria*

*Mesosuchus browni*

*Rhynchosaurus articeps*

*Proterosuchus* South African

*Proterosuchus yuani*

1->0.

29.

*Youngina*

0->1.

32.

*Rhynchosaurus*

2->1.

36.

*Uromastyx*

0->1

*Erythrosuchus*

1->0

38.

*Gephyrosaurus*

0->1

- Fused to quadrate.

45.

*Coelophysis*

*Plateosaurus*

0->”-“

47.

*Coelophysis*

*Plateosaurus*

0->”-“

51.

*Coelophysis*

*Plateosaurus*

0->”-“

*Amotosaurus*

?->0

52.

*Proterosuchus* South African

0->1.

53.

*Uromastyx*

1->0.

*Amotosaurus*

?->0

55.

*Protorosaurus*

1->0.

56.

*Trilophosaurus buettneri*

*Uromastyx*

1->0.

*Rhynchosaurus*

?->1.

60.

*Youngina*

0->?

63

*Trilophosaurus buettneri*

?->0.

64.

*Amotosaurus*

?->0.

67.

*Petrolacosaurus*

*Youngina*

*Gephyrosaurus*

0->1.

68.

*Macrocnemus bassanii*

?->0

*Amotosaurus*

?->2

69.

*Amotosaurus*

*Macrocnemus bassanii*

*Prolacerta*

*Tanystropheus*

*Youngina*

1->0

70.

*Youngina*

0->1

*Shinisaurus*

1->0.

71.

*Youngina*

0->1

73.

*Uromastyx*

1->0.

*Azendohsaurus*

0->1.

74.

*Uromastyx*

1->0.

75.

*Uromastyx*

0->1.

*Tanystropheus*

?->1

76.

*Youngina*

1->0.

*Mesosuchus*

?->0

77.

*Mesosuchus*

?->1

78.

*Amotosaurus*

?->0

79.

*Amotosaurus*

?->0.

*Langobardisaurus*

?->1.

*Macrocnemus bassanii*

0->1.

80.

*Uromastyx*

1->0.

81.

*Uromastyx*

0->1.

*Macrocnemus bassanii*

?->1.

83.

*Erythrosuchus*

*Petrolacosaurus*

*Prolacerta*

*Proterosuchus* South African

*Protorosaurus*

0->1.

*Macrocnemus bassanii*

*Proterosuchus yuani*

*Uromastyx*

?->1.

84.

*Amotosaurus*

?->0.

85.

*Langobardisaurus*

?->1.

87.

*Proterosuchus yuani*

?->0.

88.

*Proterosuchus yuani*

?->0.

89.

*Proterosuchus yuani*

?->0.

96.

*Protorosaurus*

?->0.

97.

*Shinisaurus*

*Uromastyx*

?->1.

*Macrocnemus bassanii*

*Youngina*

0->1

98.

*Uromastyx*

0->1.

*Teraterpeton*

0->2.

*Amotosaurus*

?->0.

102.

*Trilophosaurus jacobsi*

1->0.

107.

*Uromastyx*

0->1

*Proterosuchus yuani*

0->?

108.

*Mesosuchus browni*

1->0.

*Amotosaurus*

?->0.

*Proterosuchus yuani*

?->1.

112.

*Proterosuchus yuani*

0->?

113.

*Mesosuchus browni*

1->0.

114.

*Uromastyx*

0->1.

*Mesosuchus*

1->0.

115.

*Amotosaurus*

?->1.

116.

*Mesosuchus*

*Protorosaurus*

0->1.

*Euparkeria*

?->0.

*Amotosaurus*

?->1.

117.

*Batrachotomus*

*Prolacerta*

0->1.

118.

*Amotosaurus*

1->0.

119.

*Prolacerta*

0->1.

*Batrachtomus*

1->0.

*Proterosuchus yuani*

0->?

120.

*Amotosaurus*

*Protorosaurus*

?->0.

121.

*Protorosaurus*

*Youngina*

?->0.

*Proterosuchus yuani*

0->?

122.

*Youngina*

?->0

*Amotosaurus*

*Mesosuchus*

*Protorosaurus*

*Tanystropheus*

?->1

124.

*Proterosuchus* South African

0->1.

*Amotosaurus*

?->0.

125.

*Macrocnemus*

*Tanystropheus*

1->0.

*Tanytrachelos*

1->?

*Amotosaurus*

?->0.

128.

*Protorosaurus*

0->1.

*Proterosuchus* South African

*Proterosuchus yuani*

?->0.

*Amotosaurus*

*Langobardisaurus*

?->1.

129.

*Azendohsaurus madagaskarensis*

1->0.

*Amotosaurus*

?->0.

131.

*Rhynchosaurus*

*Uromastyx*

0->1.

*Tanystropheus*

1->0.

132.

*Uromastyx*

?->0

*Rhynchosaurus*

?->1

*Tanystropheus*

2->0.

133.

*Pamelaria*

?->1.

134.

*Trilophosaurus jacobsi*

?->0.

*Amotosaurus*

*Pamelaria*?->1.

135.

*Proterosuchus yuani*

?->0.

136.

*Petrolacosaurus kansensis*

*Uromastyx*

1->0.

*Prolacerta*

?->1.

*Langobardisaurus*

?->3.

141.

*Mesosuchus*

0->1.

144.

*Teraterpeton*

0->?

146.

*Uromastyx*

1->0.

*Youngina*

?->1.

147.

*Petrolacosaurus*

1->0.

*Euparkeria*

?->0.

148.

*Gephyrosaurus bridensis*

*Proterosuchus* South African

*Proterosuchus yuani*

*Shinisaurus*

*Uromastyx*

*Youngina*

0->1.

*Mesosuchus*

*Protorosaurus*

?->1

149.

*Uromastyx*

1->0.

*Youngina*

1->?

*Amotosaurus*

?->0.

150.

*Amotosaurus*

?->0.

151.

*Uromastyx*

*Youngina*

0->1.

*Amotosaurus*

?->0.

153.

*Uromastyx*

1->0.

154.

*Protorosaurus*

*Teyumbaita*

0->1.

155.

*Uromastyx*

1->0.

*Youngina*

*Teyumbaita*

?->0

156.

*Protorosaurus*

1->0.

*Youngina*

0->1

157.

*Protorosaurus*

0->?

*Proterosuchus* South African

*Youngina*

0->1.

*Macrocnemus bassanii*

1->0.

*Amotosaurus*

?->0.

158.

*Mesosuchus*

*Youngina*

0->1.

*Langobardisaurus*

?->0.

*Prolacerta*

?->1.

159.

*Langobardisaurus*

?->0

160.

*Youngina*

?->0

161.

*Youngina*

?->0

*Coelophysis*

*Plateosaurus*

1->”-“

162.

*Amotosaurus*

?->0.

163.

*Mesosuchus*

1->0.

164.

*Youngina*

1->0.

166.

*Shinisaurus*

*Uromastyx*

0->1.

*Youngina*

?->0.

167.

*Proterosuchus yuani*

0->1.

*Langobardisaurus*

1->?

*Uromastyx*

?->0

168.

*Proterosuchus yuani*

0->?

169.

*Youngina*

0->?

170.

*Proterosuchus* South African

1->?

*Uromastyx*

?->1.

172.

*Youngina*

?->0.

173.

*Proterosuchus* South African

*Proterosuchus yuani*

*Trilophosaurus buettneri*

0->1

*Euparkeria*

?->0.

*Trilophosaurus jacobsi*

?->1.

174.

*Trilophosaurus buettneri*

1->0.

*Trilophosaurus jacobsi*

?->0.

*Euparkeria*

*Macrocnemus bassanii*

?->1.

176.

*Prolacerta broomi*

0->1.

*Protorosaurus speneri*

?->1.

177.

*Amotosaurus*

?->0.

178.

*Amotosaurus*

?->1.

179.

*Amotosaurus*

*Youngina*

?->1.

180.

*Amotosaurus*

?->0.

181.

*Gephyrosaurus*

*Macrocnemus bassanii*

*Tanystropheus*

*Tanytrachelos*

*Trilophosaurus buettneri*

*Trilophosaurus jacobsi*

0->1.

*Protorosaurus*

*Youngina*

?->0.

*Amotosaurus*

*Langobardisaurus*

?->1.

182.

*Mesosuchus*

0->1.

188.

*Uromastyx*

0->1.

*Azendohsaurus madagaskarensis*

?->1.

189.

*Protorosaurus*

0->1.

*Youngina*

?->0.

194.

*Langobardisaurus*

?->1.

196.

*Protorosaurus*

*Youngina*

?->1.

*Proterosuchus yuani*

1->0.

198.

*Amotosaurus*

?->1

199.

*Youngina*

0->?

200.

*Macrocnemus bassanii*

?->0.

201.

*Amotosaurus*

?->0.

202.

*Macrocnemus bassanii*

*Mesosuchus*

*Prolacerta*

0->1.

203.

*Uromastyx*

1->?.

*Langobardisaurus*

?->0.

204.

*Amotosaurus*

?->0.

*Mesosuchus*

*Proterosuchus* South African

*Proterosuchus yuani*

*Uromastyx*

0->1.

206.

*Uromastyx*

?->1.

207.

*Tanystropheus*

0->1

*Amotosaurus*

*Macrocnemus bassanii*

?->0

*Langobardisaurus*

?->1.

208.

*Amotosaurus*

?->0.

209.

*Uromastyx*

?->0.

210.

*Gephyrosaurus*

0->1.

*Amotosaurus*

?->0.

211.

*Amotosaurus*

*Langobardisaurus*

*Uromastyx*

?->0.

215.

*Amotosaurus*

*Langobardisaurus*

?->0.

*Trilophosaurus jacobsi*

?->1.

216.

*Proterosuchus* South African

0->1.

*Amotosaurus*

?->0.

217.

*Proterosuchus* South African

*Proterosuchus yuani*

1->0.

*Amotosaurus*

?->0.

*Langobardisaurus*

?->1.

218.

*Amotosaurus*

?->0.

219.

*Gephyrosaurus*

0->?

*Youngina*

0->1.

*Uromastyx*

?->1.

221.

*Pamelaria*

*Protorosaurus*

0->1.

*Amotosaurus*

?->0.

222.

*Trilophosaurus buettneri*

1->0.

223.

*Uromastyx*

?->0.

224.

*Pamelaria*

0->1.

*Amotosaurus*

*Macrocnemus bassanii*

1->0.

225.

*Langobardisaurus*

?­->0.

226.

*Amotosaurus*

?->0.

227.

*Amotosaurus*

*Macrocnemus bassanii*

*Uromastyx*

?->0.

228.

*Uromastyx*

0->1.

*Youngina*

0->?

229.

*Protorosaurus*

*Uromastyx*

*Youngina*

?->0.

231.

*Amotosaurus*

*Protorosaurus*

*Uromastyx*

?->0.

232.

*Proterosuchus yuani*

0->1.

*Proterosuchus* South African

0->?

233.

*Pamelaria*

0->?

*Amotosaurus*

*Tanystropheus*

*Youngina*

?->0.

234.

*Protorosaurus*

?->0

235.

*Youngina*

0->?

237.

*Langobardisaurus*

?->0.

*Erythrosuchus africanus*

?->1.

238.

*Uromastyx*

1->?

*Amotosaurus*

?->0.

239.

*Uromastyx*

0->?

*Youngina*

?->0.

*Erythrosuchus*

*Proterosuchus yuani*

?->1.

241.

*Amotosaurus*

*Langobardisaurus*

?->1.

242.

*Prolacerta*

1->0/1.

*Amotosaurus*

*Langobardisaurus*

?->0.

243.

*Uromastyx*

0->1.

244.

*Erythrosuchus*

?->0.

*Amotosaurus*

?->1.

245.

*Langobardisaurus*

?->0.

246.

*Amotosaurus*

*Shinisaurus*

*Youngina*

?->0.
